# The role of maize (*Zea mays*) radicle root hairs in seedling establishment under adverse phosphorus and water seedbed conditions

**DOI:** 10.1093/aob/mcaf142

**Published:** 2025-07-09

**Authors:** Ariel Tasca, Thomas D Alcock, Gerd Patrick Bienert

**Affiliations:** Crop Physiology, TUM School of Life Sciences, Technical University of Munich, Alte Akademie 12, Freising 85354, Germany; HEF World Agricultural Systems Center, Technical University of Munich, Freising 85354, Germany; Crop Physiology, TUM School of Life Sciences, Technical University of Munich, Alte Akademie 12, Freising 85354, Germany; HEF World Agricultural Systems Center, Technical University of Munich, Freising 85354, Germany; Crop Physiology, TUM School of Life Sciences, Technical University of Munich, Alte Akademie 12, Freising 85354, Germany; HEF World Agricultural Systems Center, Technical University of Munich, Freising 85354, Germany

**Keywords:** Aquaporin, maize, nutrient deficiency, phosphorus, PHT transporter, radicle, root, root hair, water deficiency, *rth3*, *Zea mays*

## Abstract

**Background and Aims:**

A vigorous root system is crucial for maize seedling establishment. Its formation and subsequent plant performance are hindered by nutrient and water deficiency. Upon germination, maize seedlings develop primary, then seminal roots, covered with pubescent root hairs. The functions of root hairs at this developmental stage remain largely unknown. This study examined their role during phosphorus (P) and water limitations during early seedling development at the physiological, elemental and molecular level, comparing a roothairless maize mutant (*rth3*) and its isogenic wildtype (WT).

**Methods:**

Shoot and root system architecture phenotyping and elemental analysis were performed on 5-d-old *rth3* and WT plants experiencing various P- and water-deficient conditions in different growth substrates. Microscopy of root hairs and specific reverse transcription quantitative PCR of various P-nutrition regulators and aquaporins in roots were performed.

**Key Results:**

WT seedlings responded with a morphologically typical root hair elongation solely to water-reduced but not P-deficient conditions. In contrast, at the molecular level, WT and *rth3* responsively upregulated P transporters in roots upon P deficiency, while water channel transcript abundances did not change upon water limitation. Surprisingly, under these adverse seedbed conditions no differences in shoot biomass, shoot nutrient concentrations or shoot water content were detected between the WT and the roothairless mutant which additionally formed a generally shorter total root length compared to the WT. P deficiency caused the development of thicker primary roots in *rth3* and a significant increase in expression of P transporters compared to the WT.

**Conclusions:**

Germinating *rth3* seedlings showed neither disadvantages in terms of shoot vigour, nor with respect to shoot water and nutrient levels in suboptimal seedbed conditions compared to the WT, despite possessing shorter roots and no root hairs. An increase in root diameter and P-transporter expression particularly in *rth3* seminal roots may have been sufficient to physiologically compensate for the missing root hairs.

## INTRODUCTION

Germination and subsequent seeding establishment are critical growth phases in crop development, as the embryo, once protected by the seed environment, is now directly exposed to potentially ‘hostile’ environmental conditions. Successful and vigorous seedling establishment in this infant growth phase will determine plant performance, stress resistance and yield formation (Koornneef *et al*., 2002) and depends highly on a robust root system. Stunting or a suboptimal root system formation is often caused by environmental stress factors such as drought, high moisture, cold, herbicide damage, tillage compaction and mineral nutrient stress (Hossne *et al*., 2016). A retarded root system at a plant’s infancy will stunt entire further plant development (Nicola, 1998). Phenotypes of stunted and poorly developed crops in the early vegetation period are generally diagnosed as a result of below-ground ‘root causes’ (Fageria and Moreira, 2011).

Maize (*Zea mays* L.) is known to be very sensitive to water and nutrient deficiencies in the juvenile growth stage and is considered particularly inefficient at early plant establishment. To ensure high yield production, maize plants need to grow rapidly once they have germinated (Liu *et al*., 2004). However, early root development of maize is seen as a slow process after germination. Poor root growth leads to reduced nutrient uptake, creating a vicious circle where limited nitrogen and phosphate (P) absorption further restricts root growth (Lopez *et al*., 2023). In maize, it has been shown that successful stand establishment is dependent on the development of a robust root system before six leaves have unfolded (BBCH16). For instance, a reduction in total biomass and grain yield due to a limited supply of P between planting and BBCH16 is observed (Barry and Miller, 1989). Therefore, the growth stage directly preceding and nourishing maize root system formation represents a crucial phase for vigorous seedling establishment, and a vital responsibility is placed on the first developing roots scouting the soil.

In contrast to the exogenously formed primary root of most angiosperms, the primary root and first seminal roots of maize and other Poaceae are formed endogenously inside the maize seed (Hochholdinger *et al*., 2018). Once emerged, the primary root is programmed to provide anchorage as well as to secure uptake of water and nutrients in a wide range of soil seedbed conditions (Jackson and Caldwell, 1993; Chassot and Richner, 2002). During the first 2 weeks of growth, the primary (radicle) and seminal roots make up the major portion of a seedling’s root system. Shortly after the radicle penetrates the coleorhiza and enters the non-protected soil environment, the radicle and first seminal roots are pubescent, characteristically coated in a bloomy manner by numerous root hairs. While root hairs are described to be major drivers for soil anchorage and penetration as well as to secure uptake of water and nutrients (Bengough *et al*., 2016, Bienert *et al*., 2021), experiments demonstrating the biological function of those hairs in radicle roots are scarce. The mechanistic and molecular plasticity of radicle root hairs contributing to water and nutrient uptake as well as soil anchorage, but, even more importantly, the synergistic biological sum that is the health and speed of seedling establishment, are poorly understood.

### What are the roles of radicle root hairs in soil exploration and anchorage?

The physical role of maize radicle root hairs was suggested to be important for anchorage and penetration in loose seedbeds and for assisting root tips to emerge from bio-pores and explore the bulk soil, in agreement with the observation that a root hair-defective maize mutant (*rth3-3*) anchors poorly in soil (Bengough *et al*., 2016). A benchmark study demonstrated that root hairs of barley accessions with differently long root hairs were highly responsive to different soil physical conditions, such as strength, penetration resistance, compactness, soil pore sizes and soil water content (Haling *et al*., 2013).

### What are the roles of radicle root hairs in nutrient uptake and sensing?

While the conceptional understanding of plant root hair function in hydromineral nutrition seems clear, experimental evidence is restricted to a limited number of reference nutrients such as P. Studies have clearly demonstrated that root hairs are essential for the regulation of the P nutritional status in plants, and that P deficiency triggers the development and shape of root hairs (Gahoonia and Nielsen, 1998; Zhang *et al*., 2018). However, the role of radicle root hairs on nutrient acquisition during germination remains to be demonstrated. Although macro- and micronutrient reserves in maize seeds are sufficient to support seedling growth for a few days, young roots in maize seedlings are densely covered with root hairs (Nadeem *et al*., 2011, 2012*a*, 2012*b*; White and Veneklaas, 2012). Interestingly, it was shown that, on the one hand, the metabolizing of seed P was not influenced by the amount of externally supplied P, but that, on the other, P uptake started already upon emergence of the seedling radicle (Nadeem *et al*., 2011, 2012*a*). Together, this suggests that the radicle root (hair)-mediated uptake of nutrients is crucial for a quantitatively minor but essential nutritional demand of root metabolism while the total seed-stored nutrients is probably translocated to the developing shoot, a sink organ. In line with this interpretation, root hairs of only 4-d-old maize roots possess significantly different and nutrient-specific protein expression patterns in response to individual nutrient (N, P, K, Fe and Mg) deprivations (Li *et al*., 2015), though the plants’ nutrient demand is fully covered by that stored in seeds. The influence of certain mineral availabilities (B, P, Fe, Mn) on the early stages of root hair development suggest that nutritional stimuli even dominate over intrinsic developmental programmes underlying root hair differentiation studies (Müller and Schmidt, 2004; Bienert *et al*., 2021). This further pinpoints the need to clarify the role of root hairs for the hydromineral nutrition of plants.

### What are the roles of radicle root hairs in water uptake?

While nutrients and carbon sources are stored as lasting reserves in seeds, water is not. Therefore, water uptake into seedlings is constantly needed. Despite this demand for efficient water uptake during germination, and the assumption that root hairs and the rhizosphere play a key role in regulating plant water relationships, surprisingly little is known about water uptake mechanisms and decisive rhizosphere drivers, neither in maize radicles and during germination nor in mature root systems. In mature root systems, root hair traits such as length and density seem to vary in response to soil moisture variations only in newly forming but not in existing roots (Mackay and Barber, 1987). The underlying mechanisms and consequences of water (and nutrient) uptake are unknown. The question of whether root hairs relevantly contribute to water uptake has been a long-lasting one, and different studies have come to different conclusions. While the density and length of root hairs correlate with water uptake in diverse plant species (Marzec *et al*., 2015), it is reduced in root hair-less Arabidopsis and oat but not in barley and rice lines (Bienert *et al*., 2021). In barley, it was demonstrated that the beneficial effects of root hairs were evident only in dry growth seasons (Marin *et al*., 2021). This is in agreement with the observation that only in a moderately dry, but not humid, soil was more efficient water uptake observed for a WT compared to a root hair-less *brb* barley mutant (Carminati *et al*., 2017). The benefits of root hairs for the uptake of nutrients was only shown in studies concomitantly challenging maize and barley plants with a combination of water and nutrient stress, while no benefit of root hairs was seen in highly fertilized and humid soils (Brown *et al*., 2012; Klamer *et al*., 2019; Marin *et al*., 2021).

The present study experimentally addressed the hypothesis that the presence of root hairs in an emerging root system of maize seedlings is crucial to ensure vigorous shoot seedling establishment, under adverse water and P seedbed conditions. To address this hypothesis, a roothairless *rth3* maize mutant (Wen and Schnable, 1994) was compared with its isogenic B73 wildtype (WT) in control well-watered and well P-supplied (+P + H_2_O), water-reduced (+P−H_2_O), P-deficient (−P + H_2_O), or P- and water-limited double stress (−P−H_2_O) seedbed conditions. The *rth3* initiates root hair primordia but fails to elongate them properly (Hochholdinger *et al*., 2008; Li *et al*., 2016; Hey *et al*., 2017) and is therefore believed to be deprived in root hair-specific functions.

## MATERIALS AND METHODS

### Seed sterilization and germination

To investigate the function of root hairs to unfavourable growth conditions, the *Zea mays* transposon-induced roothairless mutant (*rth3*) was compared to its isogenic wildtype (WT) B73 (Hochholdinger *et al*., 2008; Wen and Schnable, 1994). The *rth3* genotype is highly homozygous due to being backcrossed to the B73 line at least eight times (Lippold *et al*., 2021).

Seeds were surface sterilized by incubation in 10 % NaClO and 1 % Tween for 10 min and subsequently rinsed at least seven times with ultrapure water. After rinsing, the seeds were soaked for 5 min in ultrapure water and then incubated for 3 h in saturated CaSO_4_. The seeds were pregerminated on filter paper soaked in saturated CaSO_4_ in sealed Petri dishes in a light-sealed environment at room temperature. Sowing took place after 3 d of pre-germination.

### Soil and substrate preparation

Two different growth media were used, a white peat-based substrate (type Fruhstorfer, HAWITA Gruppe GmbH, Vechta, Germany) (hereafter ‘peat’) and a loamy soil (hereafter ‘loam’). The following conditions were tested on all of the two substrates: with P, well-watered (+P + H_2_O); without externally supplied P, well-watered (–P + H_2_O); with P, water-reduced (+P−H_2_O) and without externally supplied P, water-reduced (–P–H_2_O). Loam was sieved to 1 mm and fertilized with liquid fertilizing solutions 2 d before sowing and subsequently dried at room temperature until use. Fertilizer in powder form was applied on the day of sowing, and the soil was sieved again to 1 mm to get a homogeneous mixture of soil and fertilizer. The nutrients applied as solutions were composed as follows (adapted from Lippold *et al*., 2021), 0.1 g N kg^−1^ soil in the form of NH_4_NO_3_, 0.18 g P kg^−1^ soil in the form of CaHPO_4_, 0.1 g K kg^−1^ soil in the form of K_2_SO_4_ and 0.05 g Mg kg^−1^ soil in the form of MgCl_2_.6H_2_O. No CaHPO_4_ was applied for −P conditions.

The peat substrate was fertilized according to Bienert *et al*. (2023) with the following adaptations: The macromix was applied as 1×, micromix as 2×, ironmix as 3× and boric acid as 6.4× concentrated in the nutrient solution. The nutrient solutions were composed as follows: 100× macromix + P: (0.21 g N kg^−1^ substrate in the form of 60 g NH_4_NO_3_ L^−1^, 0.1824 g P kg^−1^ substrate in the form of 80 g KH_2_PO_4_ L^−1^, 0.011 g K kg^−1^ substrate in the form of 6 g K_2_SO_4_ L^−1^, 0.04 g Mg kg^−1^ substrate in the form of 20 g MgSO_4_ L^−1^), 100× macromix –P: (0.21 g N kg^−1^ substrate in the form of 60 g NH_4_NO_3_ L^−1^, 0.11 g K kg^−1^ substrate in the form of 60 g K_2_SO_4_ L^−1^, 0.04 g Mg kg^−1^ substrate in the form of 20 g MgSO_4_ L^−1^), 1000× micromix (0.01 g Cu kg^−1^ substrate in the form of 13 g CuSO_4_ L^−1^, 0.01 g Zn kg^−1^ substrate in the form of 13 g ZnSO_4_ L^−1^, 0.035 g Mn kg^−1^ substrate in the form of 40 g MnCl_2_ L^−1^, 0.08 µg Mo kg^−1^ substrate in the form of 0.085 g NaMoO_4_ L^−1^), 1000× ironmix (0.0023 g Fe kg^−1^ substrate in the form of 7 g NaFeEDTA L^−1^), 100× boric acid (0.15 g B kg^−1^ substrate in the form of 1.4 g H_3_BO_3_ L^−1^). For the P-deficient treatment, no CaHPO_4_ or KH_2_PO4 was added. To achieve a similar K level in the peat substrate –P treatment, K was added in the form of K_2_SO_4_ (60 g L^−1^). Peat was limed prior to fertilization by adding 0.5 % CaCO_3_ and 0.3 % CaO and subsequently sieved to 4 mm after air drying.

### Substrate water retention curve measurement

The well-watered and water-reduced conditions were selected according to a water retention curve. Five replicates of air-dried, 4-mm sieved peat-based substrate were placed in 105-cm^3^ metal cylinders, covered by a small-pored mesh filter on the bottom of the cylinder. The cylinders were filled exactly like the soil columns to ensure the same bulk density as used within the experiments. The substrate was water-saturated and placed on a pF suction plate (ecoTech Umwelt-Messsyteme GmbH, Bonn, Germany) for the values 10, 30, 60, 150, 300 and 500 hPa (pF 1, pF 1.5, pF 1.8, pF 2.2, pF 2.5 and pF 2.7, respectively). Higher pressure application of 1000, 3000, 6000 and 12 000 hPa (pF 3, pF 3.5 and pF 4, respectively) was applied in pressure pots containing a water-soaked ceramic plate, while regularly controlling for soil–environment equilibrium by a water outlet. After reaching equilibrium, the weight of the samples was documented. To completely remove the water of the substrate, the cylinders were placed at 105 °C overnight.

### Soil and substrate growth conditions

Seedlings were grown in 50 mL light-protected brown centrifuge tubes containing 65 or 22 g of air-dried loam or peat, respectively. In every centrifuge tube, a thin layer of gravel was placed on top to prevent excessive evaporation of water. Volumetric water content was adjusted as follows: Peat + H_2_O (40 %), −H_2_O (25 %), Loam + H_2_O (22 %) and −H_2_O (18 %). Initial irrigation was done with ultrapure water (Milli-Q IQ 7000, 18.2 MΩ cm, Merck KGaA, Darmstadt, Germany) in loam. Peat was irrigated with nutrient solution the first time, and the water level was adjusted daily by weighing and adding ultrapure water. Plants were grown in a GrowBank (GroBank, CLF Plant Climatics, Germany) in 650 µm m^2^ s^−1^ illumination during a 12-h/12-h day/night cycle, having a temperature of 25 °C during the day and 18 °C during the night cycle.

To investigate how the genotypes behave in the chosen conditions at the older juvenile stage of 22 d after coleoptile emergence (DAE), WT and *rth3* plants were grown under the same above-described conditions in acrylic glass columns (25 cm height and 7 cm inner diameter) using peat substrate with the identical fertilization and irrigation plan (Bienert *et al*., 2023). The columns were filled with 350 g of air-dried, fertilized soil and irrigated up to the volumetric water content.

### Hydroponic growth conditions

Hydroponic experiments were conducted in 50-mL light-protected brown centrifuge tubes, using 50 mL of the following nutrient solutions per plant: nitrogen [2 mM Ca(NO_3_)_2_.4H_2_O], macronutrient +P (0.5 mM K_2_SO_4_, 0.1 mM KH_2_PO_4_), macronutrient −P (0.55 mM K_2_SO_4_), additional macronutrients (0.5 mM MgSO_4_.7H_2_O, 0.1 µM KCl), micronutrients [0.1 µM H_3_BO_3_, 0.25 µM MnSO_4_.H_2_O, 0.5 µM ZnSO_4_.7H_2_O, 0.2 µM CuSO_4_.5H_2_O, 0.01 µM (NH_4_)_6_Mo_7_O_24_, 0.1 mM Fe-EDTA]. The nutrient solution was changed after emergence of the plant every second day. The growth conditions were identical with previously explained experiments, except that the daytime temperature was set to 22 °C and the nighttime temperature to 18 °C. Hydroponics experiments were conducted using +P and −P conditions.

### Shoot, root and seed sampling of soil/substrate-grown plants

Plants were harvested 5 or 22 DAE. Shoot length was measured. After this, the shoots were cut and dried at 40 °C for 96 h, followed by weighing of the dry shoot. After shoot harvest, the roots were cleaned from the soil and stored in 70 % EtOH. Root scanning was done using an Epson Expression 12000XL scanner at 600 dpi and analysed using WinRHIZO Pro 2021 (Regent Instruments, Canada) software. Primary and seminal roots were analysed separately for the 5 DAE plants after being separated from the seed or all together for the 22 DAE plants. The germinated seeds and roots from 5 DAE plants were separated and dried at 80 °C for 24 h.

### Shoot and root analysis of hydroponically grown plants

The seedlings were harvested 5 DAE. Roots were stored in 70 % EtOH to perform root system architecture analysis. Shoot biomass was determined by weighing. For microscopy of the root hairs, freshly cut roots were placed in a water-filled Petri dish and observed at 1× 60-mm magnitude using a Stereo-Microscope 2× zoom factor (Zeiss SteREO Discovery.V8 with Zeiss Plan Apo S 1.0× FWD 60 mm). Root hair images were acquired with a Zeiss Axiocam 503 colour camera in Zeiss software (ZEN 2.5, blue edition).

### Root hair quantification

Plants were grown, fertilized and irrigated as described above in peat and longitudinally cut brown experimental tubes to be able to remove the root without damaging the root hairs. Seedlings at 5 DAE old were carefully cleaned from soil and rinsed with deionized water. Roots were placed into a water-filled Petri dish and observed at 1× 60-mm magnitude and 2× zoom factor using a stereo-microscope, as described above. Images for analyses were taken in a middle section of the lateral root zone and cropped exactly on the primary root boundary to only have root hairs in the images without the primary root. Every image was cropped at the same size of 450 × 600 units to avoid unintentional scaling. Root hair length measurements were performed using the Fiji (version 2.14.0/1.54f/Java 1.8.0_322) segmented line tool by manually tracing ten exemplary root hairs per image. A mean was taken from ten root length measurements. For one root, three images were analysed and subsequently a mean was calculated. This was done for three biological replicates per treatment. The mean of the biological replicates was utilized for comparison of the mean root hair length between the treatments.

### Elemental analysis

Dried shoots, and germinated and non-germinated seeds were milled to a fine powder using a ball mill (Retsch MM301). Tissue subsamples [ca. 100 or 50 mg dry weight (DW), depending on the tissue] were digested in 20 mL MARSXpress PFA digestion vessels (CEM, Kamp-Lintfort, Germany) within a 40-place turntable in a CEM MARS 6 microwave digestion system. Subsample material was digested in 2 mL of 67 % trace analysis-grade nitric acid (HNO_3_; VWR NORMATOM, VWR International GmbH, Darmstadt, Germany), 1 mL Milli-Q water (from a Milli-Q IQ 7000, 18.2 MΩ cm, Merck KGaA), and 1 mL 30 % trace analysis-grade H_2_O_2_ (Suprapur, Merck – Supelco, Merck KGaA). The microwave was set to ramp up to 65 °C over 5 min with a power of up to 100 W, at which temperature samples were held for a further 5 min. Sample temperatures were then ramped up to 190 °C over 15 min with a power of up to 900 W, at which temperature they were held for 40 min. Two operational blanks were included in each digestion run, along with duplicate samples of certified reference material (CRM) of leaf (Tomato SRM 1573a, NIST, Gaithersburg, MD, USA). Following digestion, each tube was made up to a final volume of 14 mL by adding 10 mL Milli-Q water and transferred to a 15-mL centrifuge tube (Cellstar®, Greiner Bio-One GmbH, Frickenhausen, Germany) and stored at room temperature. Tissue digestates were diluted 1-in-50 using Milli-Q water prior to elemental analysis. The concentrations of 13 elements were obtained using inductively coupled plasma-mass spectrometry (ICP-MS; PerkinElmer NexION 350D, PerkinElmer, Rodgau, Germany). Elements and specific masses measured were: B11, Na23, Mg24, P31, S34, K39, Ca44, Mn55, Fe56, Ni60, Cu63, Zn66 and Mo98. All elements with the exception of B11 were quantified using kinetic energy discrimination with helium introduced into the reaction cell at a flow rate of 4.7. B11 was measured in standard mode in which the reaction cell was evacuated. Samples were introduced using an Elemental Scientific 2DX autosampler (Elemental Scientific, Omaha, NE, USA) through a MEINHARD® concentric glass nebulizer (PerkinElmer) and into a NexION quartz cyclonic spray chamber (PerkinElmer). Internal standards were introduced via a separate line and included Re187 (10 μg L^−1^) for B11 normalization and Rh103 (10 μg L^−1^) for quantification of all other elements, each in 2.5 % trace analysis-grade HNO3. A multi-element standard solution (Multielement-Standardlösung ‘HR-ICP-MS’, Bernd Kraft, Duisburg, Germany) was used to calibrate Mg, Ca, P and K in the range 100–10 000 μg L^−1^, S in the range 1000–10 000 μg L^−1^, Na in the range 20–2000 μg L^−1^, Fe in the range 1.5–150 μg L^−1^, B, Mn, Cu and Zn in the range 0.5–50 μg L^−1^, and Ni and Mo in the range 0.05–5 μg L^−1^. ICP-MS operation and sample processing were managed using Syngistix software (PerkinElmer). Obtained concentration values were blank- and weight-normalized and are reported here in mg kg^−1^. Average elemental recovery from CRM samples ranged from 80.33 to 104.55 % for elements with certified values.

### Gene expression analysis

Primary and seminal roots were separately frozen in liquid nitrogen immediately after harvest. RNA extraction, cDNA synthesis as well as reverse transcription quantitative PCR (RT-qPCR) analysis was done as previously described (Bienert *et al*., 2023). Primer information is described in Supplementary Data Table S1. Primer design was made using the Benchling cloud-based platform.

### Statistics and graphs

Statistical analyses were performed using OriginPro 2021 software (Version 9.8.0.200). Two- or three-way ANOVAs were performed with the factors genotype, P-treatment and/or water treatment, followed by a Tukey’s honest significant differences (HSD) or Fisher test. Figures were done using R Studio (Version 4.3.3 ‘Angel Food Cake’) with the ggplot2 and ggpubr package or Excel (Version 1808; Build 10415.20025). Different letters show significant differences in the figures for *P* > 0.05.

## RESULTS

### Reduced P-uptake, water use efficiency and biomass accumulation observed in *rth3* compared to WT under well-supplied conditions after 22 DAE

**Fig. 1.**
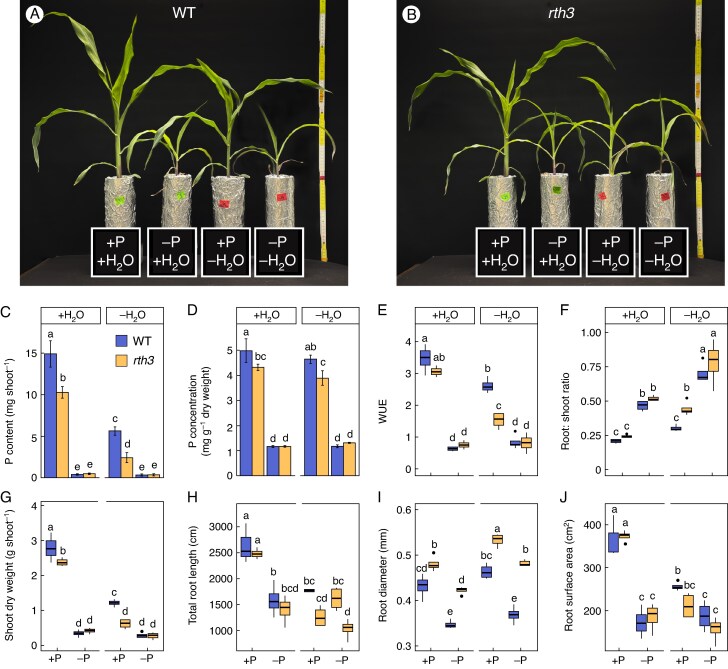
Maize plants cultivated under various water (H_2_O) and phosphorus (P) growth conditions. Representative images of 22-d-old B73 wildtype (WT) (A) and *roothairless 3* mutant (*rth3*) maize plants grown in a peat-based substrate in control well-watered and well P-supplied (+P + H_2_O), water-reduced (+P−H_2_O), P-deficient (−P + H_2_O), or on P- and water-limited double stress (−P−H_2_O) conditions. +P, 180 mg added P kg^–1^ substrate; −P, 0 mg added P kg^–1^ substrate; +H_2_O, 40 % volumetric substrate water content; −H_2_O, 25 % volumetric substrate water content. Shoot P content (C), shoot P concentration (D), water use efficiency (WUE) (E), root:shoot ratio (F), shoot dry weight (G), total root length (H), root diameter (I) and root surface area (J) of 22-d-old maize plants are displayed. Boxplots indicate the 25th and the 75th percentiles, and the horizontal line represents the median. Whiskers extend to 1.5 times the interquartile range, with outliers plotted individually. Significant differences between treatments are indicated through different lower-case letters and are calculated with a three-way ANOVA and subsequent post-hoc Tukey test (*P* ≤ 0.05, *n* = 5–6). Error bars indicate ±SD.

To assess the role of radicle root hairs of maize, B73 (WT) and *rth3* plants were compared when grown on control well-watered and well P-supplied conditions (+P + H_2_O) or on either water-reduced (+P−H_2_O) or P-limited (−P + H_2_O) conditions, or on P- and water-reduced double stress conditions (−P−H_2_O) in a peat-based substrate. At 22 DAE, plants grown in water- and P-limited conditions, either alone or in combination, were inhibited in growth compared to well-watered and well P-supplied plants (Fig. 1 A, B). Phosphorus limitation had the biggest visual effect on plant performance, which led to reduced biomass accumulation under both water availability conditions, whilst plants grown in well P-supplied but water-reduced conditions were only inferior to plants grown under well P-supplied and well-watered conditions.

To evaluate whether the lack of root hairs may have an influence on P uptake in combination with the water and P treatments, the shoot P content and concentration of 22 DAE plants were determined (Fig. 1 C, D). Under +P−H_2_O growth conditions, both genotypes showed an intermediate shoot P content and concentration compared to control (+P + H_2_O) or P-limited plants, independent of the water supply (−P + H_2_O and −P−H_2_O). Shoot P content of WT plants decreased by 62.31 % and of *rth3* plants by 76.52 % under +P−H_2_O compared to *+*P + H_2_O conditions. Under *+*P−H_2_O conditions *rth3* accumulated 57.04 % less shoot-P than the WT. In control conditions (+P + H_2_O), a 31.05 % reduced shoot P content in *rth3* plants compared to the WT was measured, a reduction that has also been detected by others (Lippold *et al*., 2021; Bienert *et al*., 2023; Hartwig *et al*., 2025). P concentration followed a similar trend as the P content, but not as substantial, where *rth3* had a 13.25 % lower P concentration than the WT under +P + H_2_O conditions and a 16.24 % lower P-concentration under −P + H_2_O conditions. As expected, a P-limited growth condition resulted in significantly reduced P content and P concentration in both WT and *rth3*.

Volumetric water contents of 40 and 25 % were chosen to represent well-watered and water-reduced conditions, respectively, according to the generated water retention curve of the peat substrate (Supplementary Data Fig. S1). Accordingly, the volumetric water content of well-watered conditions (+H_2_O) was within the plant-available water range at pF 1.9 and the water-reduced treatment (−H_2_O) was close to the plant-unavailable water range at pF 3.9. Notably, there was no variation in water use efficiency (WUE, Fig. 1 E), root:shoot ratio (Fig. 1 F), shoot DW (Fig. 1 G) as a response to P reduction between the WT and *rth3* and therewith between plants with or without root hairs. The WUE of both genotypes decreased under water-reduced (+P−H_2_O) compared to control (+P + H_2_O) conditions: 24.72 % for the WT and 50.10 % for *rth3*, respectively. Under water-reduced (+P−H_2_O) conditions, the WUE of *rth3* was 41.49 % smaller compared with the WT. Under P-limited growth conditions (−P + H_2_O and −P−H_2_O), both genotypes displayed a similar value of reduced WUE efficiency compared to well-P-supplied conditions (+P + H_2_O and +P−H_2_O), independent of the water availability of the plants (Fig. 1 E).

The root/shoot DW ratio was calculated to investigate if more plant resources had been invested in root compared to shoot development, for example to scavenge for P and H_2_O in suboptimal growth conditions (Fig. 1F). In both genotypes, P limitations caused a 54.34 % increase in the root:shoot ratio in +H_2_O and a 67.31 % increase in −H_2_O conditions compared to well-P-supplied conditions. While water reduction (+P−H_2_O) did not influence the root:shoot ratio of the WT compared to control (+P + H_2_O) conditions, *rth3* exhibited a greater investment of resources into the development of its root in such conditions (Fig. 1 F), and had a 31.70 % increased root:shoot ratio compared to the WT in water-reduced (+P−H_2_O) conditions, indicating a possible response to compensate for the lack of root hairs in the mutant. Despite the fact that its root:shoot ratio was increased, *rth3* was not able to maintain the same WUE as the WT in +P−H_2_O conditions (Fig. 1E).

The P-limited treatments generated a significant decrease of up to 87.82 % in shoot DW compared to the control *+*P + H_2_O condition in both genotypes, water independently (Fig. 1G). When grown under +P + H_2_O and +P−H_2_O conditions, the *rth3* mutant failed to accumulate the same shoot DW as the WT, experiencing a 21.71 % reduction in shoot DW. Under +P−H_2_O conditions, WT and *rth3* accumulated 60.20 and 74.16 % less shoot biomass compared to +P + H_2_O conditions, respectively (Fig. 1G).

To address further potential root surface compensating parameters in *rth3* mutants under P- and H_2_O-reduced conditions, the total root length and average root diameter were quantified. Independent of H_2_O and P availability, the root diameter of *rth3* was significantly thicker than that of the WT (Fig. 1H, I). Moreover, the *rth3* mutant reacted to +P−H_2_O or −P + H_2_O with an increase and a reduction in the root diameter, respectively, compared to the control *+*P + H_2_O condition. Neither the +P−H_2_O, nor the −P + H_2_O nor the control +P + H_2_O condition resulted in a shorter absolute total root length (TRL) of *rth3* compared to the WT under the same conditions (Fig. 1H). Independent of the growth condition, the determined total root surface area of *rth3* did not differ from the total root surface area of the WT (Fig. 1J).

To account for genotype-specific differences in shoot biomass under control +P + H_2_O conditions and to address the question of whether a lack of root hairs may even be beneficial under stress, values of the shoot P content and concentration, WUE, root:shoot ratio, shoot DW, TRL, root diameter and root surface area were normalized relative to the WT or *rth3* values obtained in the +P + H_2_O growth condition (Supplementary Data Fig. S2). However, the relative values followed the same trends as the absolute values, except for the shoot DW of *rth3* under +P−H_2_O conditions, not differing from the WT under the same conditions (Supplementary Data Fig. S2G; Fig. 1G), indicating that a lack of root hairs is not of advantage under stress conditions.

The severe phenotypic P-deficiency symptoms in combination with the extremely low P tissue concentration already 22 DAE (Fig. 1) demonstrate that the generated growth conditions constitute a P-deficient seedbed condition, probably more deficient in P than what is typically found in agricultural sites. The comparison of 22 DAE WT and *rth3* plants suggests that root hairs play a role in hydromineral nutrition of maize, as would be expected based on data from the literature on varying plant species (Marzec *et al*., 2015; Carminati *et al*., 2017; Marin *et al*., 2021) and also that the designed growth set-up is suitable to investigate the role of radicle root hairs of maize seedlings in adverse seedbed conditions.

### Primary root hair length of WT increases upon reduced water supply at the seedling stage

**Fig. 2.**
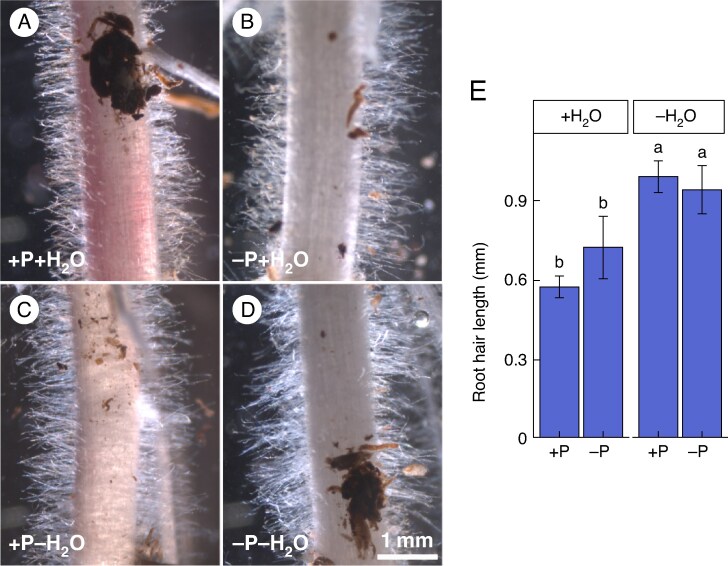
Micrographs of root hairs of the primary root (A–D) of 5-d-old B73 wildtype maize seedlings. Seedlings were grown in a peat-based substrate in control well-watered and well P-supplied (+P + H_2_O) (A), P-deficient (−P + H_2_O) (B), water-reduced (+P−H_2_O) (C), or in P- and water-limited double stress (−P−H_2_O) conditions (D). +P, 180 mg added P kg^–1^ substrate; −P, 0 mg added P kg^–1^ substrate; +H_2_O, 40 % volumetric substrate water content; −H_2_O, 25 % volumetric substrate water content. Scale bar = 1 mm. Root hair length (E) demonstrates the mean root hair length per analysed root segment, calculated based on an average of ten root hairs per biological replicate out of three biological replicates. Significant differences between the treatments are indicated through different lower case letters and are calculated with a three-way ANOVA and subsequent post-hoc Tukey test (*P* ≤ 0.05). Error bars indicate ±SD.

At 5 DAE, an increase in the length of WT primary root (PR) hairs was observed in water-reduced compared to well-watered conditions, independent of the P level (Fig. 2A–D). Root hairs of PRs were 39.86 % longer in WT plants grown under water-reduced (+P−H_2_O or −P−H_2_O), compared to control +P + H_2_O conditions (Fig. 2E). In contrast, the length of root hairs did not change as a response to P-limited conditions at either water level at 5 DAE. This visually demonstrated a water supply-dependent response of radicle root hairs already 5 DAE, suggesting a role of root hair growth features in water homeostasis but not necessarily P homeostasis.

### WT and *rth3* exhibited similar shoot water content and DW, independent of the water and P treatment at 5 DAE

**Fig. 3.**
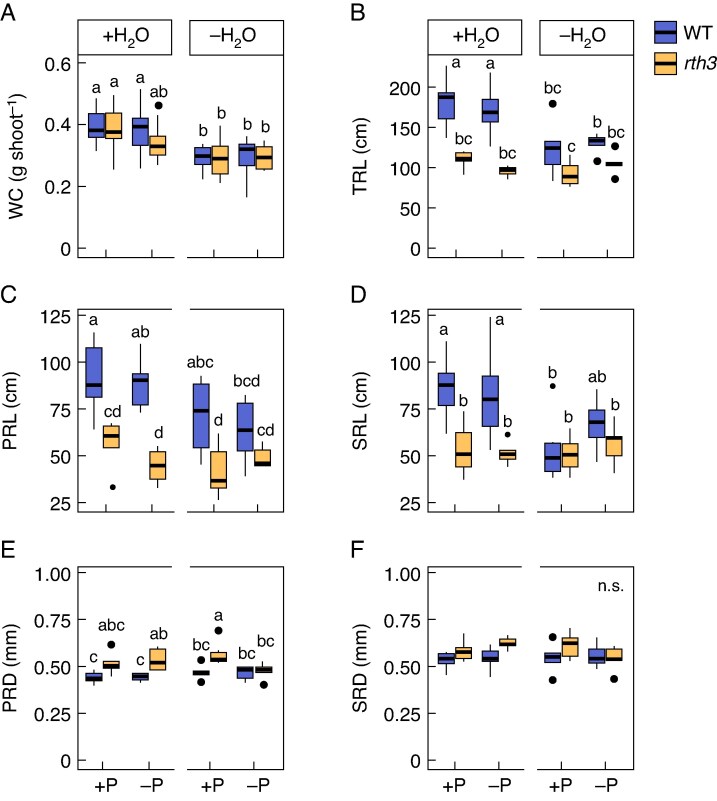
Shoot water content (WC) (A) and root system architectural traits [total root length (TRL) (B), primary root length (PRL) (C), seminal root length (SRL) (D), primary root diameter (PRD) (E) and seminal root diameter (SRD) (F)] of 5-d-old B73 wildtype (WT) and *roothairless 3* mutant (*rth3*) maize seedlings. Seedlings were grown in a peat-based substrate in control well-watered and well P-supplied (+P + H_2_O), water-reduced (+P−H_2_O), P-deficient (−P + H_2_O), or in P- and water-limited double stress (−P−H_2_O) conditions. +P, 180 mg added P kg^–1^ substrate; −P, 0 mg added P kg^–1^ substrate; +H_2_O, 40 % volumetric substrate water content; −H_2_O, 25 % volumetric substrate water content. Boxplots indicate the 25th and 75th percentiles, and the horizontal line represents the median. Whiskers extend to 1.5 times the interquartile range, with outliers plotted individually. Significant differences between the treatments are displayed by different letters and are calculated with a three-way-ANOVA and subsequent post-hoc Tukey test (*P* ≤ 0.05, *n* = 5–6).

As expected, water-reduced conditions, independent of the P supply (+P−H_2_O and −P−H_2_O), led to an average 26.53 % decrease in the water content of the shoots of both genotypes (Fig. 3A). However, comparison of the shoot water content between *rth3* and WT revealed no differences in any growth conditions at 5 DAE. Similarly, the DW of shoots of WT and *rth3* seedlings were similar in all growth conditions at 5 DAE (Supplementary Data Fig. S3A). Water treatment had a greater impact on the DW than the genotype or the P treatment, reducing the DW by 11.97 % when comparing water-reduced to well-watered conditions, independent of genotype. The shoot DW of *rth3* was 5.72 % lower compared to the WT and the P-limited treatment led to a 1.57 % reduction compared to well P-supplied conditions, independent of water (Supplementary Data Fig. S3A).

### *Rth3* possessed a shorter TRL under well-watered conditions and an increased primary root diameter compared to WT plants at 5 DAE

The TRL of the WT did not respond to the P treatments independent of the water availability but had an average 31.22 % reduced TRL in response to the water-reduction treatments (Fig. 3B). The primary root length (PRL) of the WT was 33.02 % lower under −P−H_2_O conditions compared to the WT PRL under +P + H_2_O conditions and the seminal root length (SRL) of the WT was 38.04 % lower under +P−H_2_O conditions compared to the WT SRL under +P + H_2_O conditions (Fig. 3C, D). The TRL of *rth3* was unaffected by both water and P treatments, but was on average 41.41 % shorter under well-watered conditions, independent of P, compared to the WT under the same conditions, while TRL in *rth3* was similar to the WT under water-reduction, independent of P (Fig. 3B). Similar trends were observed for PRL (Fig. 3C) and SRL (Fig. 3D).

In contrast to having a shorter TRL in well-watered conditions, independent of the P supply (+P + H_2_O and −P + H_2_O), *rth3* developed an about 11.88 % thicker PR in −P + H_2_O and +P−H_2_O growth conditions (Fig. 3E). No differences in seminal root diameter (SRD) were detected between the genotypes, regardless of the water and P supply (Fig. 3F). The total root surface area of *rth3* was on average 33.64 % smaller compared to WT plants under well-watered conditions, independent of P. No differences in the total root surface area between the genotypes were seen under water reduction, independent of P (Supplementary Data Fig. S3B).

In addition to the experiments performed on peat, WT and *rth3* plants were additionally exposed to +P + H_2_O, +P−H_2_O, −P + H_2_O and −P−H_2_O growth conditions on a loam soil and to +P + H_2_O and −P + H_2_O in a hydroponic experiment. In loam, *rth3* developed a shorter TRL than the WT in all conditions except for when comparing *rth3* and the WT under +P−H_2_O conditions (Supplementary Data Fig. S3). In contrast to the findings in peat, a larger SRD of the *rth3* mutant compared to the WT was observed under −P + H_2_O conditions, while no genotypic differences in PRD were observed. In the hydroponics cultivation system, no differences in TRL were seen but the PRD of *rth3* was 13.92 % larger under P-limited conditions compared to the WT while the SRD was always larger in *rth3* independent of the treatment, on average by 15.14 %, when compared to the WT (Supplementary Data Fig. S4).

### No genotypic differences in shoot P content were observed under P-limited conditions at 5 DAE

**Fig. 4.**
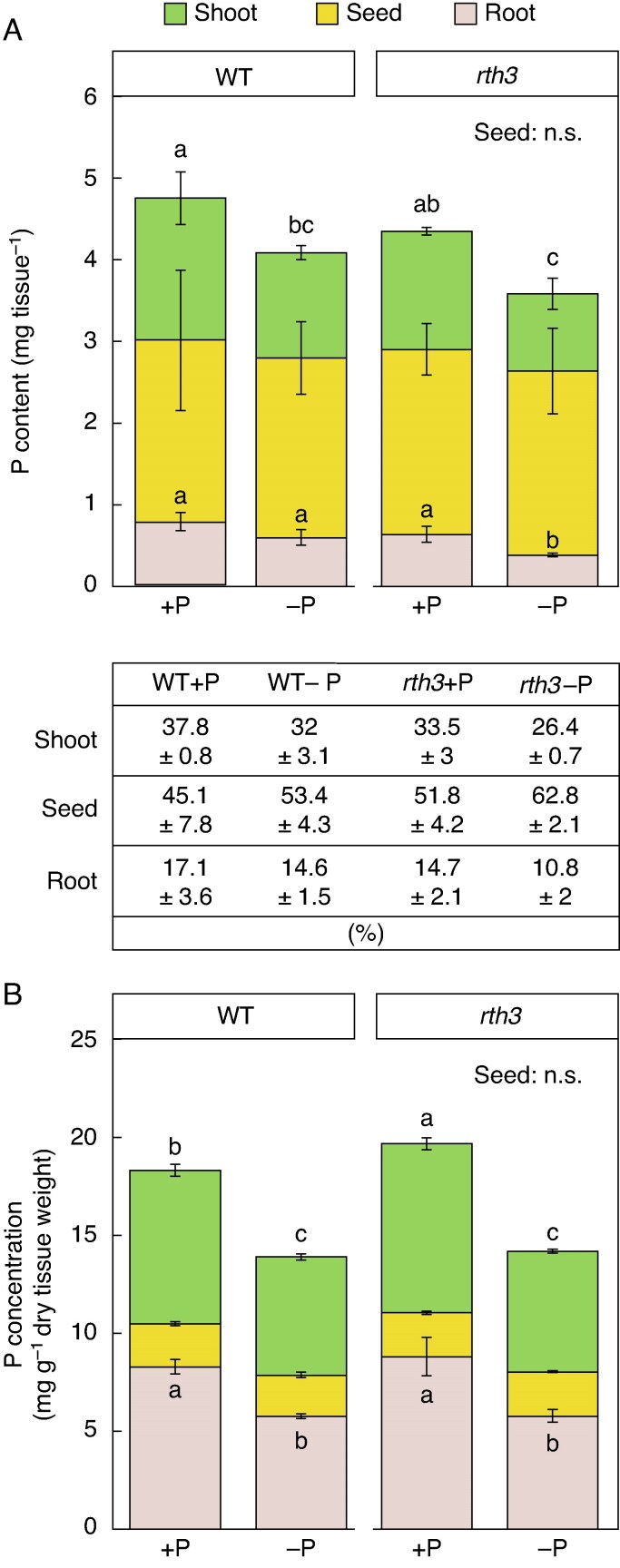
Elemental analysis of 5-d-old B73 wildtype (WT) and *roothairless 3* mutant (*rth3*) maize seedlings grown in hydroponics in either P-supplied (+P, 0.1 mM P) or P-reduced (−P, 0 mM P) conditions. P content (A) and P concentration (B) of shoot (green), root (reddish) and seed (yellow) tissue of WT and *rth3* mutant seedlings are shown. Distribution of P between shoots, seeds and roots are described as a percentage (%) in the table below the stacked bar chart in A. Significant differences between treatments are demonstrated through different lower-case letters and are calculated with a two-way-ANOVA and subsequent post-hoc Fisher test (*P* ≤ 0.05). Error bars indicate ±SD. The analysis is based on three replicates of a pool of three to five plants.

We assessed whether nutrient and in particular P acquisition differed between WT and *rth3* mutants at the seedling stage. First, element contents and concentrations of *rth3* and WT seeds were determined, in order to exclude a seed nutrient-driven advantageous germination and vigour of one over the other genotype. Concentrations and contents for various macro- and micronutrients, including P, of the non-germinated seeds did not differ between the genotypes (Supplementary Data Fig. S5). When comparing shoot P content and concentration of WT and *rth3* seedlings at 5 DAE, no differences between the genotypes were detected either on well- or reduced-P-supplied seedlings and independent of the water supply. Meanwhile, both genotypes displayed a reduction (of about 35.88 %) in shoot P content when grown on P-limited conditions (Supplementary Data Fig. S6). To determine whether a potential reduced P uptake of *rth3* roots due to the absence of root hairs is compensated for by an increased usage of seed-stored P in *rth3*, in a manner that could explain the similar P shoot contents of *rth3* and WT seedlings, P distribution patterns of roots, shoots and seeds of *rth3* and WT plants were quantified in hydroponically grown seedlings 5 DAE. In these conditions, WT seedlings had a total of 4.74 mg P per plant under P-sufficient (+P) and 4.09 mg P per plant under P-limited (−P) conditions (including shoot, seed and root tissue), while the *rth3* mutant accumulated 4.35 mg P per plant under P-sufficient (+P) and 3.59 mg P per plant under P-limited (−P) conditions. A decrease of P in the *rth3* root was observed under P-limitation (−P) as the *rth3* root had less biomass, with 51.11 % less P observed than the P-sufficient (+P) WT root and 41.19 % less P than the P-sufficient (+P) *rth3* root (Fig. 4A). On average, not considering the genotypes and treatments, 32.4 % of total seedling P was detected in shoots and 14.3 % in the roots, while 53.3 % remained in the seeds. A non-significant, but consistent over the repeated experiments, trend of less P in the shoots and roots of the WT and *rth3* mutant was observed under P-limitation. Interestingly, the seed P content proportion to shoot and root within the seedling was on average 9.65 % higher under P-limitation, not considering the genotypes, indicating that less seed-P might be relocated under unfavorable conditions and that P-distribution within the seedlings was adapted to the P-deficient environment. The shoot P concentration of *rth3* under P-sufficient (+P) conditions was 10.41 % higher than the WT shoot P concentration under the same conditions, while no difference between the genotypes was found in the other analysed tissues (seed and root) under identical conditions (Fig. 4B). The observed increase of shoot P concentration under P-sufficient (+P) conditions in *rth3* compared to the WT under the same conditions was not the case when grown on peat substrate (Supplementary Data Fig. S6B).

### P-transporters were upregulated in *rth3* seminal roots under −P + H_2_O conditions compared to WT seminal roots under the same conditions and compared to WT and *rth3* seminal roots under control (+P + H_2_O) conditions

**Fig. 5.**
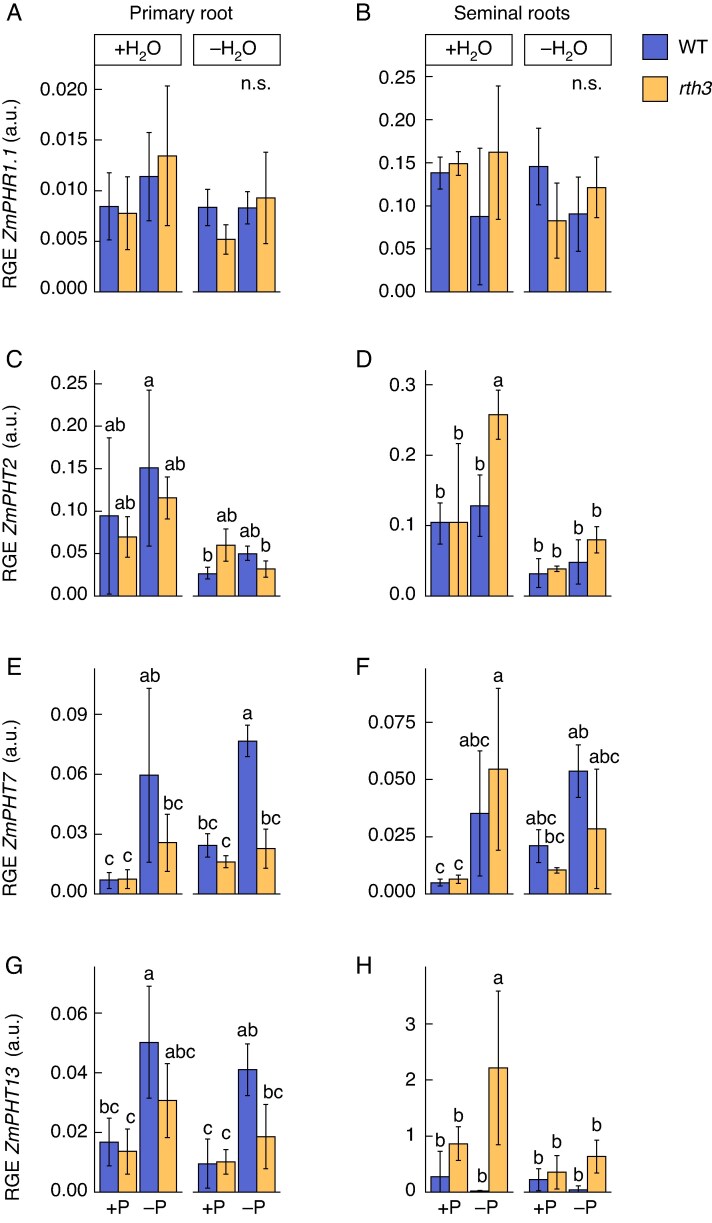
Relative gene expression (RGE) of phosphorus (P) starvation response genes in primary (PR) (A, C, E, G) and seminal roots (SR) (B, D, F, H) of 5-d-old B73 wildtype (WT) and *roothairless 3* mutant (*rth3*) maize seedlings. Relative expression of the transcription factor *ZmPHR1.1* (A, B) and root-located P-transporters *ZmPHT2* (C, D), *ZmPHT7* (E, F) and *ZmPHT13* (G, H) of WT (blue) and *rth3* (yellow) seedlings grown on a peat-based substrate in control well-watered and well P-supplied (+P + H_2_O), water-reduced (+P−H_2_O), P-deficient (−P + H_2_O), or on P- and water-limited double stress (−P−H_2_O) conditions were determined by quantitative polymerase chain reaction (qPCR). +P, 180 mg added P kg^–1^ substrate; −P, 0 mg added P kg^–1^ substrate; +H_2_O, 40 % volumetric substrate water content; −H_2_O, 25 % volumetric substrate water content. Relative expression was calculated relative to the geometric mean of the reference genes *ZmGAPDH* and *ZmACT1*. Significant differences between the treatments are indicated by different lower-case letters and calculated with a three-way ANOVA and subsequent post-hoc Tukey test (*P* ≤ 0.05). Error bars indicate ±SD; n.s. = not significant; *n* = 3.

An explanation for similar shoot P levels in WT and *rth3* seedlings grown on low soil-P availability, despite the lack of root hairs and the smaller total root surface of *rth3* plants, may be (1) that molecular compensation mechanisms ensure an increased P uptake efficiency of *rth3* mutants, for instance by increasing P transporter expression levels, or (2) that plants do not yet suffer from P-limiting conditions 5 DAE due to sufficient seed P reserves. A qPCR approach was conducted to test the primary and seminal root expression of genes known to respond to P limitation and commonly denominated as P-starvation response (PSR) genes. The MYB-like transcription factors *ZmPHR1;1* and *ZmPHR1;2* are critical regulators of PSR (Tian *et al.,* 2024). However, no differences in the relative gene expression (RGE) of *ZmPHR1.1* and *ZmPHR1.2* were observed as a reaction to the P-limited treatment, either in the PR or the SRs of WT and *rth3* mutants at 5 DAE (Fig. 5A–B; Supplementary Data Fig. S7A–B). The high-affinity P-transporters *ZmPHT2*, *ZmPHT7* and *ZmPHT13* are expressed in maize roots (Nagy *et al*., 2006) and were chosen as representative candidate P-transporter genes to test the linkage between root hairs and P-limitation at the seedling stage (Fig. 5C–H). Both *ZmPHT7* and *ZmPHT13* were significantly increased in the PR by up to 10.9-fold and up to 3-fold in WT plants exposed to P-limitations (−P + H_2_O and −P−H_2_O) compared to well P-supplied (+P + H_2_O and +P−H_2_O) plants, while no significant up-regulation of these genes was observed in *rth3* plants in the same growth conditions in the PR (Fig. 5E). In contrast, the relative gene expression of *ZmPHT2* in *rth3* SRs increased under −P + H_2_O conditions by 2.8-fold when compared to both WT and *rth3* seedlings grown in control +P + H_2_O conditions and an increase of the transcript level of *ZmPHT7* in *rth3* SR by 8.4-fold was observed when compared to *rth3* +P + H_2_O conditions. Additionally, the transcript level of *ZmPHT13* in *rth3* SRs was significantly increased by 123.24-fold in −P + H_2_O compared to the WT and by 2.6-fold compared to +P + H_2_O *rth3* seedlings (Fig. 5H). These results indicate a possible attempt to compensate for the lack of root hairs in the *rth3* mutant by an increase in gene expression of the P-transporters *ZmPHT2* and *ZmPHT13*, while this increase appeared to be specifically related to −P + H_2_O conditions.

### Aquaporin gene expression is similar between *rth3* and WT primary and seminal roots

**Fig. 6.**
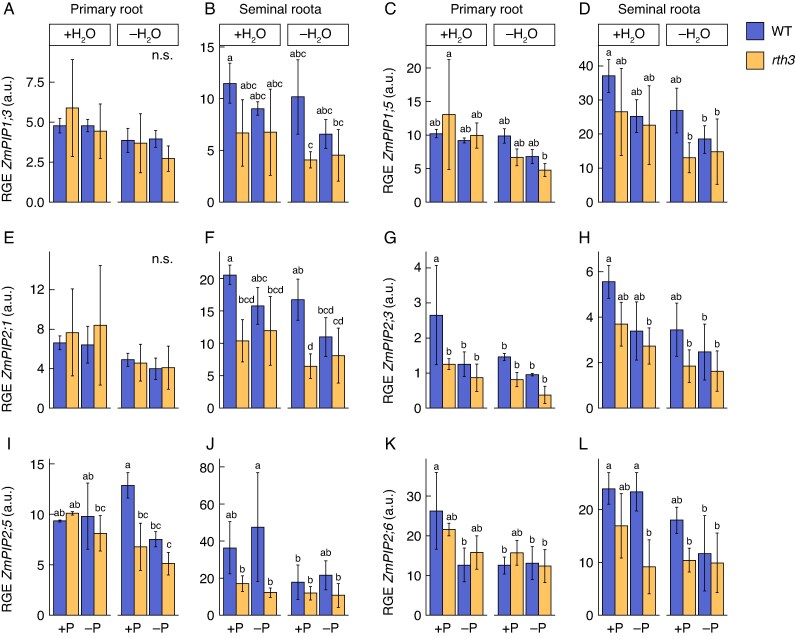
Relative gene expression (RGE) of aquaporin-encoding genes in primary (PR) and seminal (SR) roots of 5-d-old B73 wildtype (WT) and *roothairless 3* mutant (*rth3*) maize seedlings. Relative expression of *ZmPIP1;3* (A, B), *ZmPIP1;5* (C, D), *ZmPIP2;1* (E, F), *ZmPIP2;3* (G, H), *ZmPIP2;5* (I, J) and *ZmPIP2;6* (K, L) of WT (blue) and *rth3* (yellow) seedlings grown in a peat-based substrate in control well-watered and well P-supplied (+P + H_2_O), water-reduced (+P−H_2_O), P-deficient (−P + H_2_O), or in P- and water-limited double stress (−P−H_2_O) conditions were determined by quantitative polymerase chain reaction (qPCR). +P, 180 mg added P kg^–1^ substrate; −P, 0 mg added P kg^–1^ substrate; +H_2_O, 40 % volumetric substrate water content; −H_2_O, 25 % volumetric substrate water content. Relative expression was calculated relative to the geometric mean of the reference genes *ZmGAPDH* and *ZmACT1*. Significant differences between the treatments are marked by different lower-case letters and calculated with a three-way ANOVA and subsequent post-hoc Tukey test (*P* ≤ 0.05). Error bars indicate ±SD; n.s. = not significant; *n* = 3.

An analysis of molecular responses to water reduction was conducted by examining the expression of seven representative plasma membrane intrinsic proteins (PIPs) expressed in the maize root system (Hachez *et al*., 2006) and two tonoplast intrinsic proteins (TIPs) in the PRs and SRs (Fig. 6A–L; Supplementary Data Fig. S7C–H). All of the analysed Aquaporins (AQPs), except *ZmPIP2;6*, appeared to be more abundantly expressed in SRs compared to PRs. In PRs, a reduced RGE of six of the nine tested AQPs was quantified under water reduction compared to the WT +P + H_2_O treatment (all except *ZmPIP1*, *ZmPIP2;1* and *ZmPIP2;5*), while the expression of these AQPs was lower in *rth3* in the SRs compared to the WT under +P + H_2_O conditions. No differences in the RGE of *ZmPIP1;5* and *ZmPIP2;1* between the different treatments and genotypes were identified. Under water-reduced (+P−H_2_O) conditions in the PR, the expression of *ZmPIP2;5* was 47.26 % lower in the *rth3* mutant compared to the WT. The expression of *ZmPIP2;5* decreased 45.43 % in the *rth3* mutant under double stress conditions (−P−H_2_O) relative to the WT in control (+P + H_2_O) conditions. For *ZmPIP2;3*, the RGE in *rth3* under +P + H_2_O conditions was 52.55 % lower in the PR than in the WT under the same conditions. Similar behaviour was observed in the SRs, where *rth3* had a generally lower *ZmPIP2;3* than the WT under every condition. In PRs and SRs, the RGE of *ZmPIP1;1*, *ZmPIP2;3*, *ZmPIP2;6*, *ZmTIP2;1* and *ZmTIP2;3* in *rth3* remained unchanged in +P−H_2_O compared to +P + H_2_O conditions (Fig. 6; Supplementary Data Fig. S7).

## DISCUSSION

### Maize plant performance is positively influenced by the presence of root hairs within the first 3 weeks of growth upon germination

First, performance of the genotypes was investigated in a peat-based substrate during the first 3 weeks of growth to evaluate the impact of root hairs on regulation of the plants’ water and P status during the juvenile growth stage under suboptimal water and P growth conditions. At 22 DAE, *rth3* had a significant lower shoot P content and concentration than the WT when well supplied with P in the peat substrate. This is in agreement with previous studies, demonstrating that *rth3* possessed lower shoot P contents than the WT in loamy and sandy soils (Lippold *et al*., 2021; Bienert *et al*., 2023; Hartwig *et al*., 2025). These observations support the general assumption that root hairs are important for the P-nutritional status of maize plants under sufficient P supply. Under highly P-limited growth conditions, shoots of WT and *rth3* (Fig. 1A, B) showed substantial growth and biomass reduction, a strong anthocyanin colouring and senescence of the leaves, all being typical symptoms of P-deficiency. P-deficiency in maize starts at a critical threshold of below 2.6 mg P g^−1^ DW for juvenile plants with a height between 40 and 60 cm (Reuter *et al*., 1997). In the P-limited conditions, the shoot P concentration was below this critical threshold in both genotypes (Fig. 1D), namely below 1.3 mg P g^−1^ DW, demonstrating the high efficacy of the deficiency treatment used in this study and the inability of the WT to outperform *rth3* under severe P-limited growth conditions. In a high P-demanding species such as maize (Bergmann, 1993), such nutrient-limited seedbed conditions are expected to challenge seedling establishment particularly as, nutritionallly, maize is a very inefficient seedling establisher. Due to an increased root diameter compared to the WT, as already observed in other studies (Lippold *et al*., 2021; Vetterlein *et al*., 2022; Bienert *et al*., 2023), *rth3* developed a root surface area similar to that of the WT (Fig. 1J). Consequently, the insufficient ability to accumulate the same amount of shoot biomass and shoot P as the WT, when well P-supplied, derived probably from the lack of root hairs in *rth3*. Accordingly, at 22 DAE, under water-reduced growth conditions (+P−H_2_O), *rth3* accumulated a significantly lower shoot biomass and possessed a significantly lower WUE compared to the WT (Fig. 1E, G). The *rth3* mutant reacted to water limitations, compared to well-watered conditions, with a significantly increased root to shoot biomass ratio and a thicker root diameter. No such morphological compensation response was observed in the WT, although the severe water deficit significantly limited plant growth in both genotypes compared to the well-watered conditions (Fig. 1G). As concluded from the P deficiency experiment, also under water limitations, *rth3* probably failed to develop the same shoot biomass as the WT due to the lack of root hairs.

### Shoot performance and water content do not differ between *rth3* and WT seedlings germinating in water-reduced growth conditions

The dormancy of most seeds is broken by moist environmental conditions and the process of germination is marked by rapid water uptake (Shu *et al*., 2015; Macovei *et al*., 2017). In contrast to nutrients such as P, water is a limiting resource of seedlings as it is not stored in the seed. This suggests that radicle root hairs significantly contribute to water uptake directly after germination. In agreement with this assumption, an elongation of root hairs 5 DAE was noted as a morphological response to water reduction in the WT (Fig. 2E). An elongation of root hairs upon water deficit in maize and other plant species in order to increase the surface area facilitating water uptake was observed by others and is well described in mature plants (Péret *et al*., 2011; Li *et al*., 2017; Gilroy and Jones, 2000; Tsang *et al*., 2024). Choi and Cho (2019) analysed the physiological role of root hairs in *Arabidopsis thaliana* seedlings, demonstrating that root hairs significantly improved the water-holding capacity of the root, where long-haired genotypes showed greater water retention than the short-haired genotypes. In accordance with the literature, the authors suggest that root hairs promote liquid water surface adherence, resulting in an increase of water menisci around the root and subsequently boost water uptake. The observed elongation of root hairs of the WT in response to water limitations (+P−H_2_O versus +P + H_2_O) might help the plant to maintain its water acquisition ability in dry soils. This assumption is supported by the experiments with 22-DAE-old plants, where clear benefits (better WUE and an increase of shoot DW) of the WT under water-reduced conditions compared to the *rth3* mutant became clear. Surprisingly, no benefits of the WT despite its capability to form root hairs were seen on shoot growth parameters such as shoot DW gain or shoot height at the seedling stage (5 DAE) compared to the roothairless *rth3* mutant under water-reduced germination growth conditions (Fig. 1A; Supplementary Data Fig. S1A) though the water limitations significantly reduced the water content in both genotypes significantly (Fig. 3A). In contrast to the 22-DAE-old *rth3* plants, at 5 DAE, also no morphological compensation mechanisms were detected at the root level of *rth3* plants in response to water limitations compared to the well-watered growth conditions. The *rth3* mutants remained non-reactive towards different water availabilities: TRL, PRL and SRL as well as primary and seminal root diameters remained similar compared to the well-watered situation. Strikingly, in the WT, water limitations resulted in a reduced TRL compared to the well-watered conditions, leading to a root surface area comparable to the *rth3* mutant in the same growth condition. Together, this suggests that during germination and shortly thereafter, the lack of root hairs in *rth3* compared to the WT did not result in any growth disadvantage in our experimental set-up with respect to water homeostasis. A major role of root hairs in water uptake remains controversial (Cai and Ahmed, 2022). In *A. thaliana*, the roothairless mutant *AtNR23* showed a strong reduction in water absorption and decreased drought tolerance (Tanaka *et al*., 2014). A study conducted in the roothairless barley mutant *brb* demonstrated the importance of root hairs in facilitating water uptake by decreasing the drop in matrix potential at the root–soil interface in rapidly transpiring plants (Carminati *et al*., 2017). Duddek *et al*. (2023) demonstrated that maize root hair shrinkage in dry soil conditions masks the potential benefits of root hairs, while Cai *et al*. (2021) mention no significant effects of root hairs on water uptake and soil-plant hydraulic conductance. While no visually measurable shrinkage of root hairs in the WT was visible at 5 DAE in water-reduced growth conditions on the used peat substrate, no positive effect of root hairs for seedling establishment in water-reduced growth conditions was noted in the WT compared to *rth3*.

In addition to the lack of identified macroscopic differences between the WT and *rth3* mutant at the shoot level exposed to water-reduced growth conditions, also no differences in the expression of water-channelling AQP-encoding genes were detected at the molecular level. None of the *AQPs* in the primary or seminal roots, neither *PIPs* nor *TIPs* (Fetter *et al*., 2004, Hachez *et al*., 2006), showed a differential expression between the two genotypes, also possessing a similar root system size, in the water-reduced growth conditions. The transcript abundance of six of the nine tested *AQPs* decreased in primary roots of the WT upon water reduction. Similarly, a recent study on root responses to water deficit in maize seedlings showed a transcript reduction of *PIPs* when exposed to severe drought (Protto *et al*., 2024), suggesting that the seedlings in this study were experiencing similarly strong stress conditions. Hartwig *et al*. (2025) described a similar expressional trend in 3-week-old plants that had been exposed to drought, speculating that the downregulation of AQPs might be an adaptive response to protect the plant from further water loss. In contrast to the general downregulation of *AQPs* in the WT in water-reduced versus well-watered germination growth conditions, expression of *ZmPIP1;1*, *ZmPIP2;3*, *ZmPIP2;6*, *ZmTIP2;1* and *ZmTIP2;3* genes in *rth3* remained unchanged. The non-reactiveness of the *rth3* mutant towards water deficit observed at the macroscopic physiological level was therewith also observed at the molecular level. The lower expression of a few AQPs in *rth3* compared to the WT (Fig. 5) may be attributed to the lack of root hairs in the mutant, given that the genes are expressed in root hairs, as previously suggested (Hartwig *et al*., 2025).

### Shoot performance and shoot P content do not differ between *rth3* and WT seedlings germinating in P-limited growth conditions

Despite the difference in root anatomical traits, such as the lack of root hairs, a significant shorter TRL and a significant smaller root surface area, *rth3* was able to produce the same shoot DW as the WT and contained the same shoot P content and concentration as the WT under P-limited (−P + H_2_O) growth conditions. In addition to this, and contrary to findings in the literature, no elongation of root hairs was detected in the WT when grown on the P-limited peat substrate. This suggests that the lack of root hairs may not be of importance for proper seedling establishment under P-deficiency. In the study by Brown *et al*. (2012), the physiological consequences of different root hair phenotypes, namely no, short and long root hairs, were analysed in barley plants. The no-root-hair phenotype produced more shoot DW compared to the short- or long-root-hair phenotype after 7 d while having an increased TRL under P-deficient conditions. These results may indicate that the roothairless barley mutant was investing resources that have been available due to the missing formation of root hairs into a longer TRL, allowing an increase in shoot biomass compared to the short- or long-root-hair phenotypes. It is tempting to speculate that at the seedling stage the *rth3* mutant is more ‘cost-efficient’, as no or little investment of resources towards root hair production and a longer root system is made, allowing the *rth3* seedlings to perform at a similar level as the WT in terms of biomass and P-accumulation. Comparing the quantitative P distribution between *rth3* and WT seedlings identified no differences between these genotypes (Fig. 4; Supplementary Data Fig. S3). Additionally, no clear morphological reactions of roots or shoots towards the P-limited environment were observed within the first 5 DAE (Fig. 3; Supplementary Data Fig. S1). The distribution of P was adapted to the amount of P supplied in the nutrient solution. Although not significantly, but repeatedly throughout the experiment repetitions, both genotypes seemed to save seed P resources in P-limited growth conditions, suggesting that seedlings were adapting to the P-deficient environment in a similar manner (Fig. 4). At 5 DAE, seedlings are probably still sufficiently supplied with P by seed P reserves, as maize seed P supplies were shown to feed maize seedlings up to 15 d after sowing (Nadeem *et al*., 2011). Even if WT and *rth3* seedlings exhibited an already lower total plant P content in P-limited conditions, both in peat and hydroponics, compared to well P-supplied conditions and although P uptake from the environment takes place at this early developmental stage (Nadeem *et al*., 2011, 2012*a*, 2012*b*), the main P source for the growing seedlings may be P in the form of seed-stored phytate. Nevertheless, maize seedlings have been demonstrated to take up P from soils already 4 d after sowing (Nadeem *et al*., 2011), agreeing with our results, which quantified a reduction of the shoot P contents in both genotypes in P-limited growth conditions. As unexpectedly neither morphological nor nutritional differences were detected between shoots of *rth3* and the WT, grown on P-limited conditions, a gene expression analysis was performed to assess whether possible compensatory mechanisms at the molecular level may be mediated by PSR-related genes in the *rth3* mutant. Expression of the PSR transcription factors *ZmPHR1.1* and *ZmPHR1.2* was not affected by the P-limited environment, nor was it different between the two genotypes. It is known that, although being a key regulator of the P starvation response, *PHR1* genes are rather non-reactive in P-limited environments (Shibata and Sugimoto, 2019), which might explain why there was differential expression detected within this study. Similar to our study, P-transporter expression profiles, except for *ZmPHT7*, between P-limited *rth3* and WT had been studied previously (Nagy *et al*., 2006). Here, we detected a significant upregulation of *ZmPHT2* and *ZmPHT13* under P-limited (−P + H_2_O) conditions in the SRs of *rth3* compared to the SRs of the WT, which indicated that *rth3* might compensate for the lack of root hairs by upregulation of these P-transporters primarily in SRs, but not in PRs. This is in agreement with the observation that the main P uptake during the early seedling stage is mediated by SRs and their emerging lateral roots (Holz *et al*., 2024). In future studies, it will be interesting to specifically quantify the expression level of *ZmPHT2* and *ZmPHT13* in root hairs to investigate whether these transporters are root hair-located and therefore whether *rth3* needs to compensate via an upregulation of these transporters in SRs. P deficiency significantly induced the expression of *ZmPHT7* and *ZmPHT13* in the WT compared to well P-supplied growth conditions at 5 DAE (Fig. 5). In agreement with the developmentally early expressional upregulation of *PHT* transporter-encoding genes observed in our study, at a time point when P reserves in seeds are quantitatively still sufficient to supply the demands of seedlings, a proteomic analysis of maize root hairs developed in nutrient-depletion growth conditions detected an upregulation of a root hair-located P-transporter also already in 4-d-old seedlings (Li *et al*, 2015).

### The *rth3* genetic background impacts on root but not on shoot architecture

Interestingly, the *rth3* mutant developed a substrate- and treatment-dependent shorter TRL compared to the WT already at 5 DAE. In contrast to the observed shorter TRL, an elongation of the fine root system may be speculated, as this may compensate for a reduced root surface area in *rth3* mutants due to the lack of root hairs. However, such a shorter root phenotype of *rth3* was additionally documented in several other studies in older maize plants exposed to different growth conditions (Lippold *et al*., 2021; Bienert *et al*., 2023; Hartwig *et al*., 2025). It might be speculated that this phenotype can be explained by pleiotropic effects of the missing *RTH3* gene, which codes for a COBRA-like protein involved in cell-wall biosynthesis and expansion (Hochholdinger *et al*., 2008) or due to missing signals derived from root hair properties in response to the soil environment regulating other root growth features. The latter hypothesis is supported by the observation that in hydroponic culture where no physical obstacles acted on root growth and where water and nutrients were not limited, no differences in the TRL were detected in the *rth3* mutant compared to the WT (Supplementary Data Fig. S2). Similarly to *rth3*, the short root hair mutant *roothairless2* (*rth2*), representing an *RTH3*-independent gene mutation, formed a reduced root biomass independent of the abiotic stress soil growth conditions (Klamer *et al*., 2019). Together these observations in *rth2* and *rth3* maize mutants may suggest that root hair functions contribute to root system architecture development in a soil environment in which water and nutrients may not be freely available, even if well fertilized and irrigated. Alternatively, root hairs may positively impact on root growth signalling touch or contact with solid soil particles. Such a hypothesis matches previous studies demonstrating that root hairs of the radicle and the primary root system appeared to be beneficial in facilitating soil penetration as well as soil anchorage of seedlings (Haling *et al*., 2013; Bengough *et al*., 2016). As no benefits of root hairs at the very early seedling stage for shoot growth and nutrient levels as well as water shoot levels were identified in this study, one initially dominating root hair function may reside in soil anchorage and penetration features. At the molecular level, developmentally early formed root hairs may contribute to the sensing of the plant’s soil environment and prepare for water and nutrient uptake functions when seed stores become limiting and seedling growth accelerates. In the future, it will be necessary to characterize root hairs in detail at this early growth stage at the molecular level to further assess their function.

## CONCLUSION

The impact of maize root hairs seems negligible for P and water uptake within the first 5 d upon coleoptile emergence at the seedling stage in both adverse and favourable seedbed conditions. However, root hairs seemed to impact on subsequent root system architecture development already at this early growth stage. Moreover, the contribution of root hairs to nutrient and water acquisition at this early developmental stage was obviously demonstrated by the observations that (1) root hairs morphologically elongate in response to water-reduced conditions in order to increase the root surface and (2) the *rth3* mutant developed an increased root diameter and significantly upregulated the expression of P transporters (*ZmPHT2* and *ZmPHT13*) in response to P-limited seedbed conditions. Subsequently, the consequences of the lack of root hairs were then physiologically visible at 22 DAE when *rth3* plants failed to accumulate similar biomass and shoot P levels compared to the WT under well P-supplied, well-watered or water-reduced conditions.

## Supplementary Material

mcaf142_Supplementary_Data
