# Supplementary material for: The role of maize (Zea mays) radicle root hairs in seedling establishment under adverse phosphorus and water seedbed conditions
Source: Ann Bot. 2025 Jul 9;136(5-6):1115–30. doi: 10.1093/aob/mcaf142 (PMC12682883; doi:10.1093/aob/mcaf142)
Supplement: mcaf142_Supplementary_Data [file mcaf142_supplementary_data.zip › Supplementary Information_Tascaetal_revision.pdf]

**Supplementary Information**

**Title**:

The role of maize (*Zea mays* L.) radicle root hairs in seedling establishment under adverse phosphorus and water seedbed conditions

**Authors**:

Ariel Tasca[1,2], Thomas D. Alcock[1,2], Gerd Patrick Bienert[1,2]

**Institutional addresses**:

[1]Crop Physiology, TUM School of Life Sciences, Technical University of Munich, Alte Akademie 12, 85354 Freising, Germany

[2]HEF World Agricultural Systems Center, Technical University of Munich, 85354 Freising, Germany

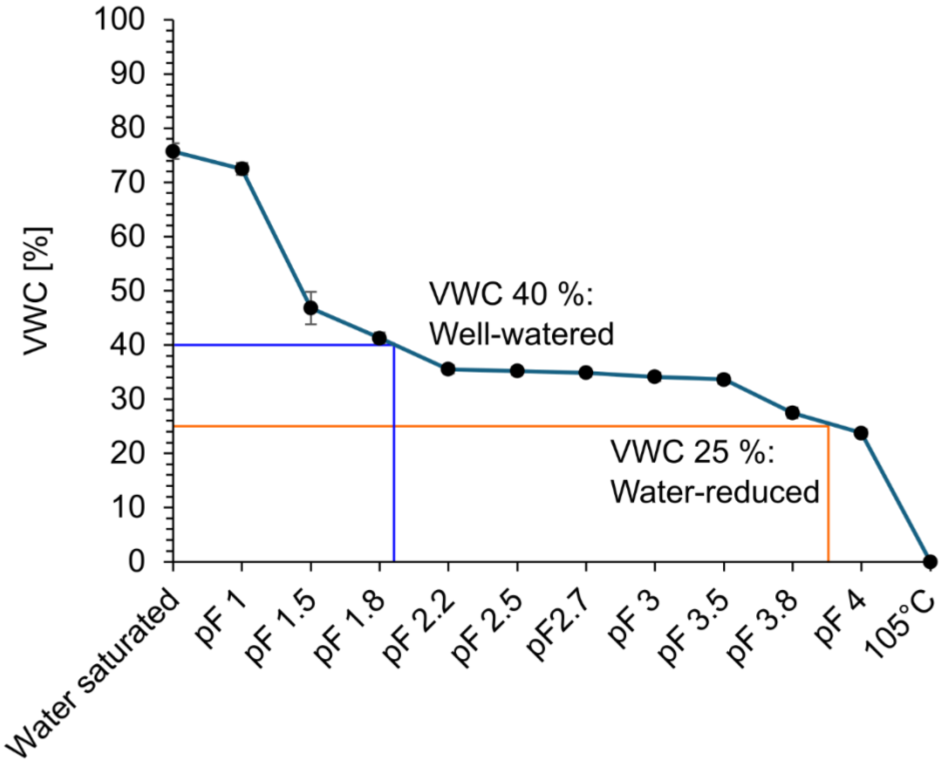


**Figure S1:** Water retention curve of the peat-based substrate sieved to 4 mm. Well-watered (+$H_2O$) conditions (VWC: 40 %) have a pF value of 1.9, while water-reduced (-$H_2O$) conditions (VWC: 25%) reach a pF value of 3.9. One dot on the curve represents the mean from n = 5 replicates ± SD. VWC = Volumetric water content.

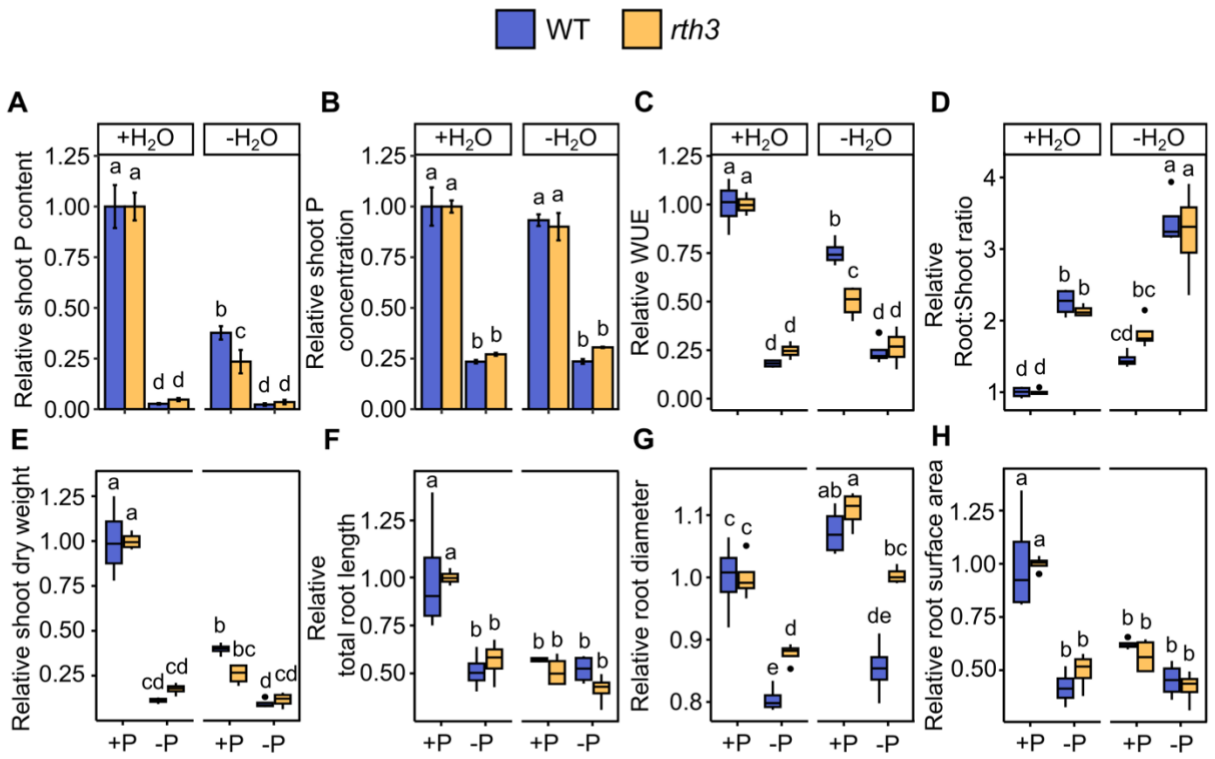


**Figure S2**: Relative shoot P content (A), relative shoot P concentration (B), relative WUE (C), relative Root:shoot ratio (D), relative shoot dry weight (E), relative total root length (F), relative root diameter (G) and relative root surface area (H) of 22-day-old B73 (WT) and roothairless 3 mutant (*rth3*) maize plants grown in a peat-based substrate in control well-watered and well P-supplied (*+P+$H_2O$*), water-reduced (*+P-$H_2O$*), P-deficient (*-P+$H_2O$*), or on P- and water-limited double stress (*-P-$H_2O$*) conditions. (+P, 180 mg added P/kg substrate; -P, 0 mg added P/kg substrate; +$H_2O$, 40% volumetric substrate water content; -$H_2O$, 25% volumetric substrate water content). The raw data values are normalized relative to their genotypes' control condition (*+P+$H_2O$*) using the following equation: $X_n = X_t \cdot \frac{1}{X_c}$ where $X_n$ represents the normalized value, $X_c$ the control condition and $X_t$ the treated condition. Boxplots indicate the 25th and the 75th percentiles, the horizontal line represents the median. Whiskers extend to 1.5 times the interquartile range, with outliers plotted individually. Significant differences between treatments are indicated through different letters and are calculated with a three-way-ANOVA and subsequent post-hoc Tukey Test ($p \leq 0.05$, n = 5-6). Error bars indicate ± SD.

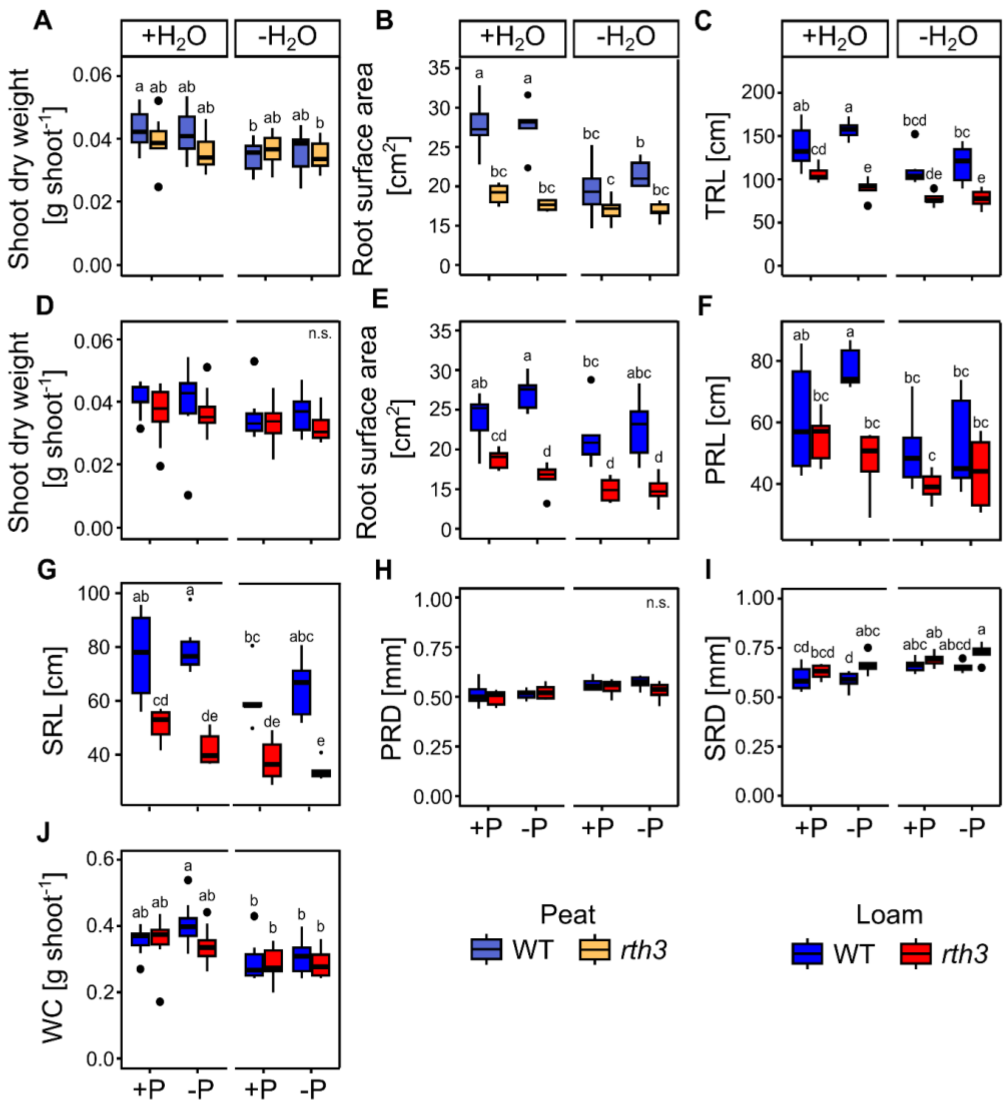


**Figure S3:** Shoot dry weight (A, D), root surface area (B, E), total root length (C), primary root length (PRL) (F), seminal root length (SRL) (G), primary root diameter (PRD) (H), seminal root diameter (SRD) (I), and shoot water content (WC) (J) of 5-day-old B73 wildtype (WT) and roothairless 3 mutant (*rth3*) maize seedlings are shown. Seedlings were grown on a peat-based substrate (A, B) or on a loamy soil (C-J) in control well-watered and well P-supplied ($+P+H_2O$), water-reduced ($+P-H_2O$), P-deficient ($-P+H_2O$), or on P- and water-limited double stress ($-P-H_2O$) conditions (+P, 182.4 (loam) or 180 (peat) mg added P/kg substrate/soil; -P, 0 mg added P/kg substrate/soil; $+H_2O$, 40% (peat) or 22% (loam) volumetric substrate water content; $-H_2O$, 25% (peat) or 11% (loam) volumetric substrate water content. Blue-toned colors depict the WT; red (loam) and yellow (peat) -toned colors depict the *rth3* mutant. Boxplots indicate the 25th and the 75th percentiles, the horizontal line represents the median. Whiskers extend to 1.5 times the interquartile range, with outliers plotted individually. Significant differences between the treatments are indicated by different letters and are calculated with a three-way-ANOVA and subsequent post-hoc Tukey Test ($p \leq 0.05$, $n = 5$-$6$).

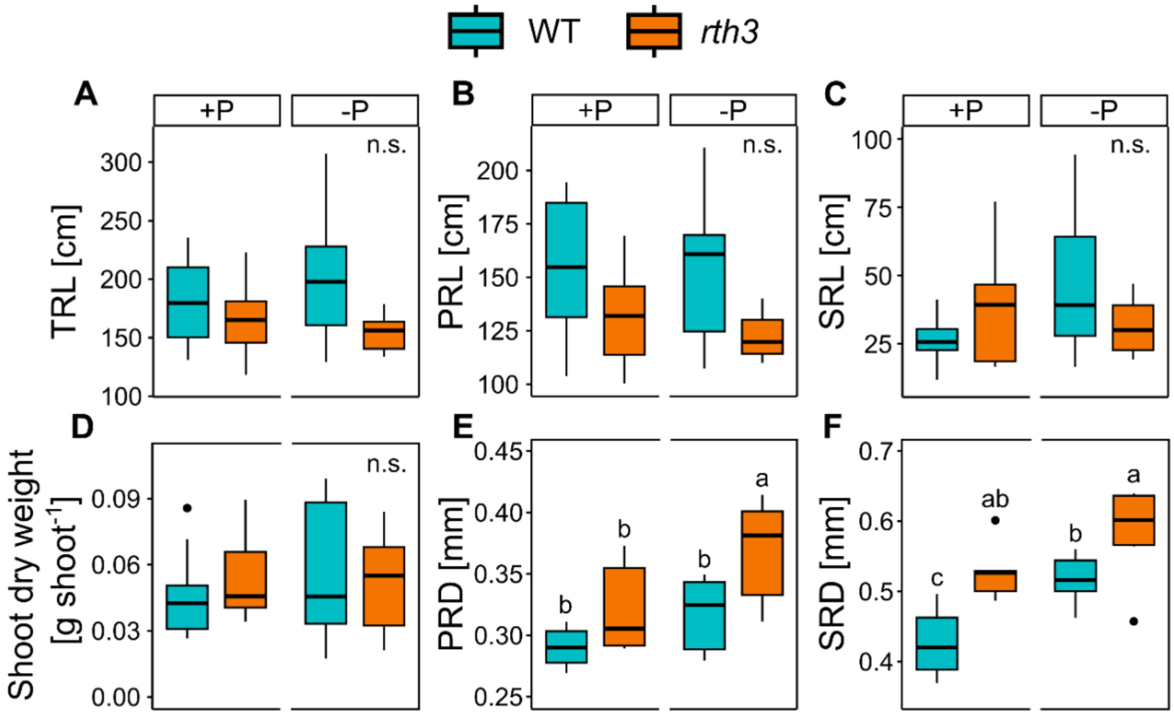


**Figure S4:** Total root length (TRL) (A), primary root length (PRL) (B), seminal root length (SRL) (C), shoot dry weight (D), primary root diameter (PRD) (E) and seminal root diameter (SRD) (F) of 5-day-old B73 wildtype (WT; turquoise-colored box plots) and roothairless 3 (*rth3*; orange-colored box plots) maize seedlings are displayed. Maize seedlings were grown in phosphorus (P) sufficient (+P; 0.1 mM P) or P-deficient (-P; 0 mM P) conditions in a hydroponic culture. Boxplots indicate the 25th and the 75th percentiles, the horizontal line represents the median. Whiskers extend to 1.5 times the interquartile range, with outliers plotted individually. Significant differences between the treatments are indicated by different letters and are calculated with a three-way-ANOVA and subsequent post-hoc Tukey Test ($p \leq 0.05$, n = 5-6).

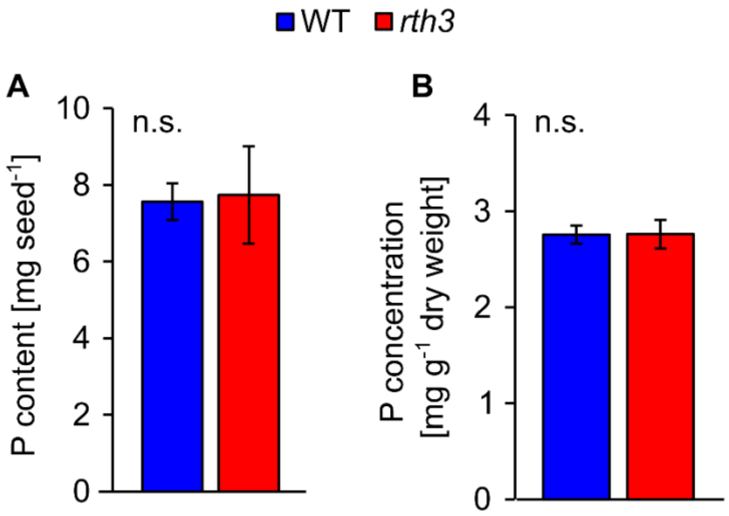


**Figure S5:** Elemental analysis of non-germinated B73 wildtype (WT; blue bar charts) and roothairless 3 mutant (*rth3*; red bar charts) maize seeds. Seed P-content (A) and seed P concentration (B) are shown. Bars represent means (± SD) of measurements of n = 10 seeds. Significant differences are calculated with a Student's t-test. Error bars indicate the ± SD. n.s. = not significant.

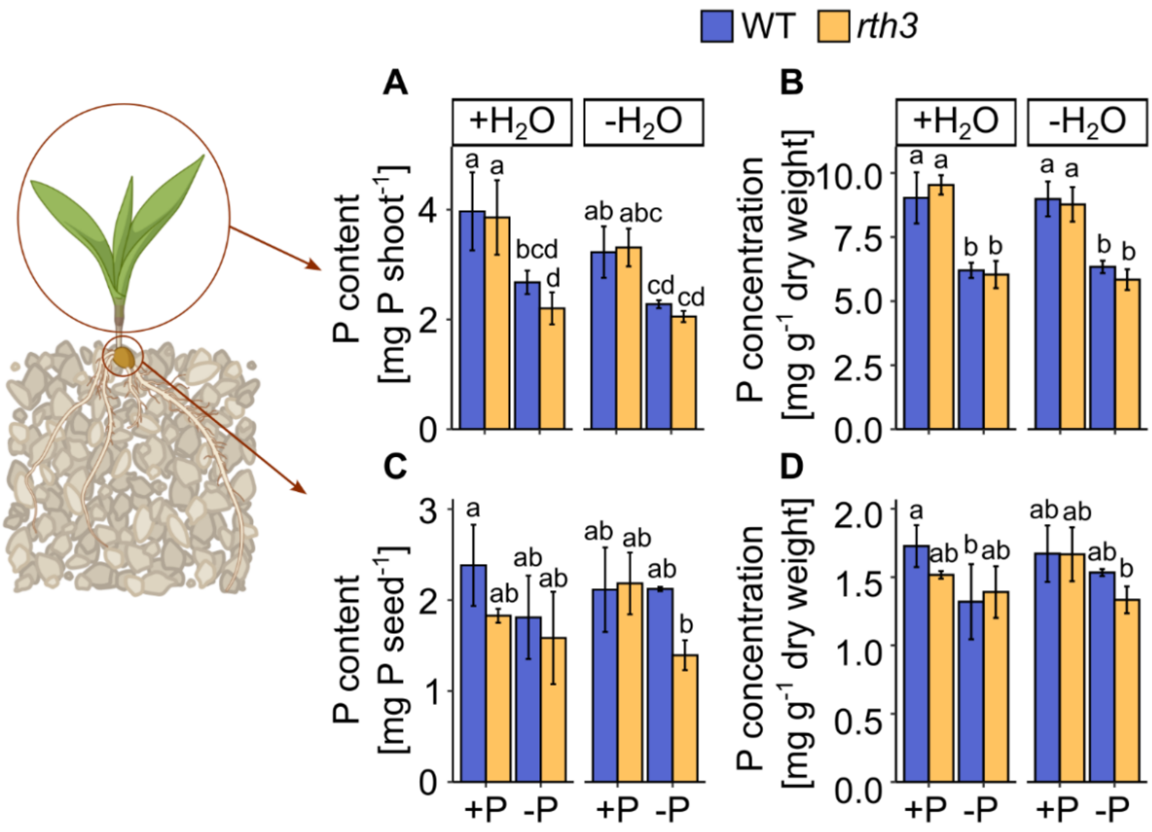


**Figure S6:** Elemental analysis of 5-day-old B73 wildtype (WT) (blue) and roothairless 3 mutant (*rth3*) (yellow) maize seedlings. Seedlings were grown on a peat-based substrate in control well-watered and well P-supplied (*+P+H2O*), water-reduced (*+P-H2O*), P-deficient (*-P+H2O*), or on P- and water-limited double stress (*-P-H2O*) conditions (+P, 180 mg added P/kg substrate; -P, 0 mg added P/kg substrate; +$H_2O$, 40% volumetric substrate water content; -$H_2O$, 25% volumetric substrate water content). Shoot P content (A), shoot P concentration (B), germinated seed P-content (C) and germinated seed P concentration (D) of 5-day-old WT and *rth3* mutant seedlings are shown. Significant differences between the treatments are demonstrated through different letters and are calculated with a three-way ANOVA and subsequent post-hoc Tukey Test ($p \leq 0.05$). Error bars indicate ± SD. Analysis was performed on 3 replicates of a pool of 3 plants. Created in BioRender. Tasca, A. (2025) https://BioRender.com/pzkjxas

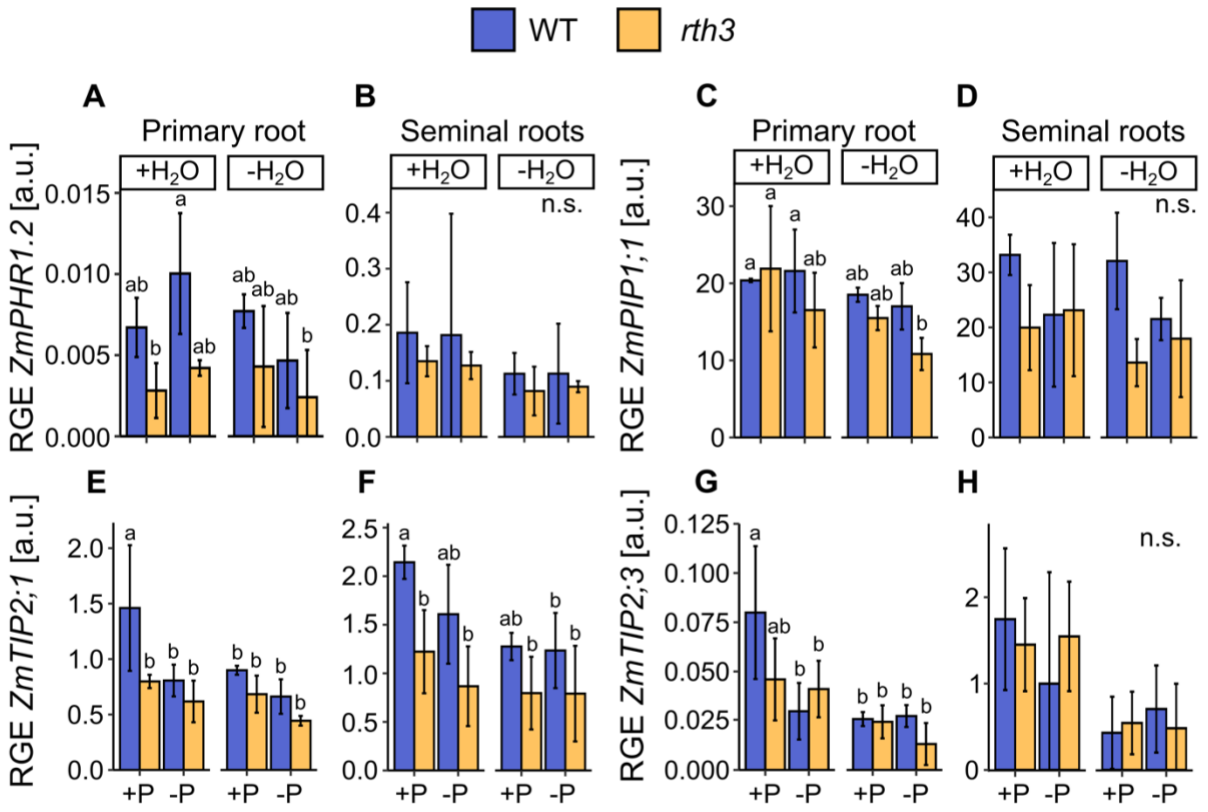


**Figure S7:** Relative gene expression (RGE) of primary root (PR) and seminal root (SR) located transcription factor *ZmPHR1.2* (A, B) and the water channeling aquaporins *ZmPIP1;1* (C, D), *ZmTIP2;1* (E, F), *ZmTIP2;3* (G, H) of 5-day-old B73 wildtype (WT) (blue) and roothairless 3 mutant (*rth3*) (yellow) maize seedlings are displayed. Seedlings were grown in a peat-based substrate in control well-watered and well P-supplied (*+P+$H_2O$*), water-reduced (*+P-$H_2O$*), P-deficient (*-P+$H_2O$*), or in P- and water-limited double stress (*-P-$H_2O$*) conditions (+P, 180 mg added P/kg substrate; -P, 0 mg added P/kg substrate; +$H_2O$, 40% volumetric substrate water content; -$H_2O$, 25% volumetric substrate water content). RGE was determined by quantitative polymerase chain reaction (qPCR) and calculated relative to the geometric mean of the reference genes *ZmGAPDH* and *ZmACT1*. Significant differences between the treatments are indicated by different letters and calculated with a three-way ANOVA and subsequent post-hoc Tukey Test ($p \leq 0.05$). Error bars indicate the ± SD. n.s. = not significant. n = 3.

**Table S1:** Primer sequences used in this study

| Gene ID | Gene Name | Direction | Sequence | Reference (if available) |
|---|---|---|---|---|
| Zm00001eb348450 | *ZmAct1* | fw | GCCCTGCTGTATGAAATGGA | [1] |
| | | rv | AAAGGAACCAGCTAAAAGCAAAC | |
| Zm00001eb173410 | *ZmGAPDH* | fw | TTGTTTCCCTTCCTGCTACC | [1] |
| | | rv | AAACTGCAACCTCACCACAAG | |
| Zm00001eb015750 | *ZmPHR1.1* | fw | AGTGACTTATGGAAAAGAAACAGA | |
| | | rv | GGCAGAATAGGCAAAGAGGAT | |
| Zm00001eb306220 | *ZmPHR1.2* | fw | AGAGTGTTTTGATGGAGAGAGAA | |
| | | rv | GAAGAGAACCTTACAGACGGAG | |
| Zm00001eb222510 | *ZmPHT1* | fw | GCCTTCACCTTCTTCTTCGC | [2] |
| | | rv | CCGTCTTGCTCCTGTCCTG | |
| Zm00001eb047070 | *ZmPHT2* | fw | CATTGTCACGCTCGTCATCT | |
| | | rv | GGTGGAGTTGAAGTGGTCGT | |
| Zm00001eb038740 | *ZmPHT7* | fw | AGTCCAAGGGCAAATCCCTG | [3] |
| | | rv | GCACTGTGCGGTTGTTGTAG | |
| Zm00001eb087740 | *ZmPHT12* | fw | TCGTTGCCGTGGTGGTATCAG | |
| | | rv | AGCATGAGAACGATCCGCCAG | |
| Zm00001eb004100 | *ZmPHT13* | fw | GAGCGCCACCATCATGTCCGAG | |
| | | rv | GATGGTGCCGAAGAGGATGCCG | |
| Zm00001eb074210 | *ZmPIP1;1* | fw | CCCCTACTATGTTACGTGGAGTTC | [4] |
| | | rv | GCGGCATATTACACAATTGGTA | |
| Zm00001eb186900 | *ZmPIP1;3* | fw | GGTTCCCGTATCCTTTTATGC | [4] |
| | | rv | AATCCAGCTGATAGATAAACCCAC | |
| Zm00001eb190950 | *ZmPIP1;5* | fw | CGTCTCATCTGCTATTGTTCGT | [1] |
| | | rv | GGACTAAACAGAGAAGTCACAAAGT | |
| Zm00001eb306380 | *ZmPIP2;1* | fw | CGGGTCGCCTTTTTTTTG | [4] |
| | | rv | CCCTTGAGAGTCACGACATGA | |
| Zm00001eb185300 | *ZmPIP2;3* | fw | AGTACGTGCTGAGAGCCAGC | [4] |
| | | rv | CGTACGTATCTACACTTGGATCGAT | |
| Zm00001eb077130 | *ZmPIP2;5* | fw | TGTCGTCGTTGGTTGCCT | [4] |

| | | | | |
|---|---|---|---|---|
| | | rv | CACAACAATCACACTAG CTTGGAA | |
| Zm00001eb306400 | *ZmPIP2;6* | fw | CAGGCGTTTGTGAGTTTG TG | [1] |
| | | rv | TCACTTGCATTCGTCCAT CT | |
| Zm00001eb186570 | *ZmTIP2;1* | fw | GTGTATGCATGTATGTGC CAAT | [5] |
| | | rv | CCAAGAAATCCATGCGT AAACA | |
| Zm00001eb429750 | *ZmTIP2;3* | fw | TACCGTCAGAGGGAGTG GGAGA | [5] |
| | | rv | CGAAGAGCAGCGTGGCA ATGA | |

[1] Heinen, R.B., Bienert, G.P., Cohen, D., Chevalier, A.S., Uehlein, N., Hachez, C., Kaldenhoff, R., Le Thiec, D., Chaumont, F., 2014. Expression and characterization of plasma membrane aquaporins in stomatal complexes of Zea mays. Plant Mol. Biol. 86, 335–350. https://doi.org/10.1007/s11103-014-0232-7

[2] Liu, F., Xu, Y., Jiang, H., Jiang, C., Du, Y., Gong, C., Wang, W., Zhu, S., Han, G., Cheng, B., 2016. Systematic Identification, Evolution and Expression Analysis of the Zea mays PHT1 Gene Family Reveals Several New Members Involved in Root Colonization by Arbuscular Mycorrhizal Fungi. Int. J. Mol. Sci. 17, 930. https://doi.org/10.3390/ijms17060930

[3] Ganther, M., Vetterlein, D., Heintz-Buschart, A., Tarkka, M.T., 2021. Transcriptome sequencing analysis of maize roots reveals the effects of substrate and root hair formation in a spatial context. Plant Soil. https://doi.org/10.1007/s11104-021-04921-0

[4] Hachez, C., Moshelion, M., Zelazny, E., Cavez, D., Chaumont, F., 2006. Localization and Quantification of Plasma Membrane Aquaporin Expression in Maize Primary Root: A Clue to Understanding their Role as Cellular Plumbers. Plant Mol. Biol. 62, 305–323. https://doi.org/10.1007/s11103-006-9022-1

[5] Su Y, Liu Z, Sun J, Wu C, Li Y, Zhang C and Zhao L (2022) Genome-Wide Identification of Maize Aquaporin and Functional Analysis During Seed Germination and Seedling Establishment. Front. Plant Sci. 13:831916. doi: 10.3389/fpls.2022.831916
